# Supplementary material for: Thioester-Containing Protein TEP27 in Culex quinquefasciatus Promotes JEV Infection by Modulating Host Immune Function
Source: Int J Mol Sci. 2025 Dec 3;26(23):11727. doi: 10.3390/ijms262311727 (PMC12692319; doi:10.3390/ijms262311727)
Supplement: Supplementary file 1 [file ijms-26-11727-s001.zip › ijms-3943100-supplementary.pdf]

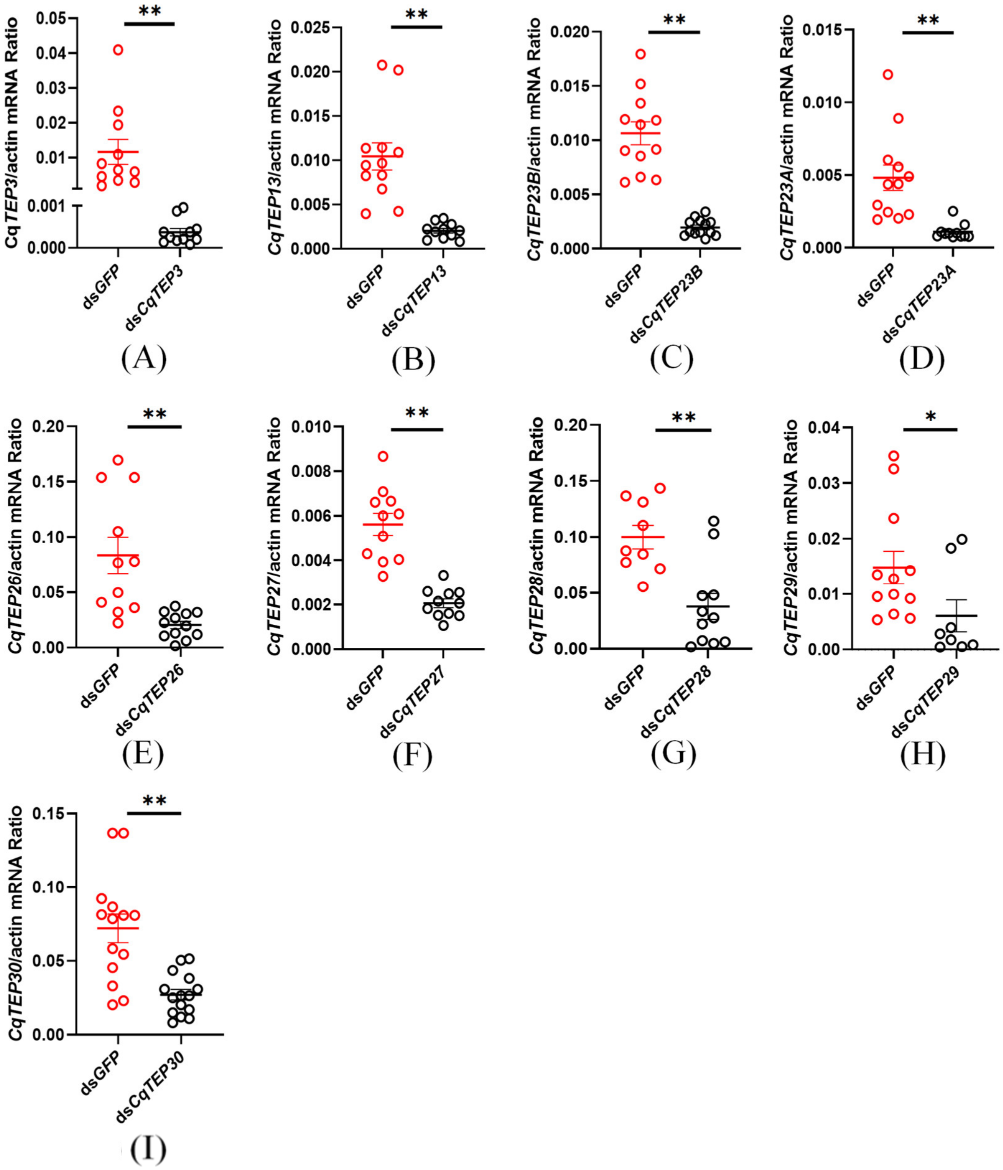

Figure S1. DsRNA-mediated knockdown efficiency of nine *Cq*TEP genes.

(**A–I**) Relative mRNA levels of *Cq*TEP genes in *C. quinquefasciatus* at 6 days post-dsRNA injection (targeting individual *Cq*TEPs) and 3 days post-JEV challenge, quantified via RT-qPCR ($n = 12$). Statistical significance is indicated as follows: ** $p < 0.01$; * $p < 0.05$.

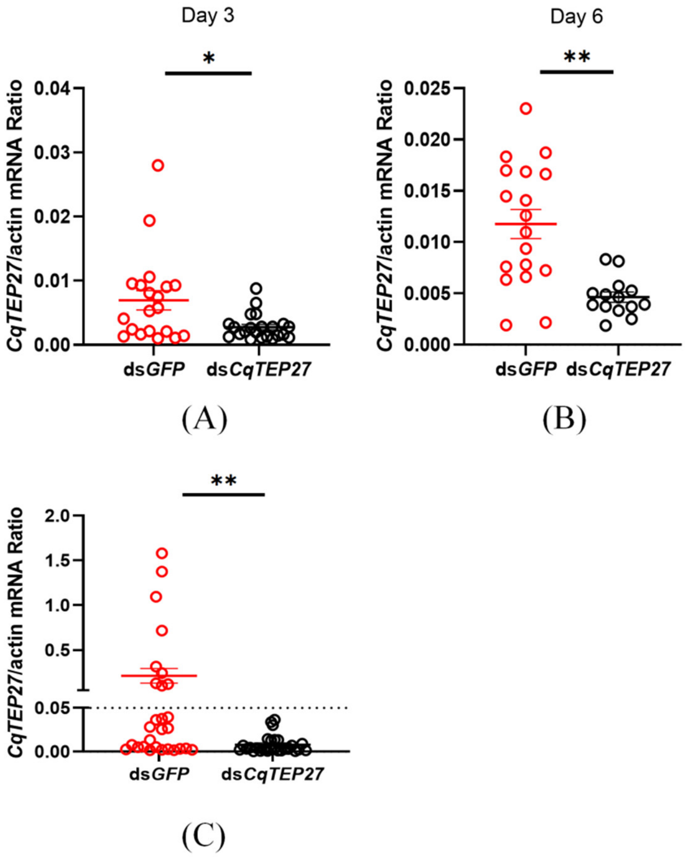

Figure S2. DsRNA-mediated *CqTEP27* silencing efficiency.

(**A**–**C**) Relative mRNA levels of *Cq*TEP*27* measured by RT-qPCR at3 dpi (**A**), (*dsGFP*, $n = 20$; *dsTEP27*, $n = 22$), 6 dpi (**B**) (*dsGFP*, $n = 18$; *dsTEP27*, $n = 15$) and (**C**) ($n = 40$) 8 days post-blood-meal. Statistical significance is indicated as follows: ** $p < 0.01$; * $p < 0.05$.

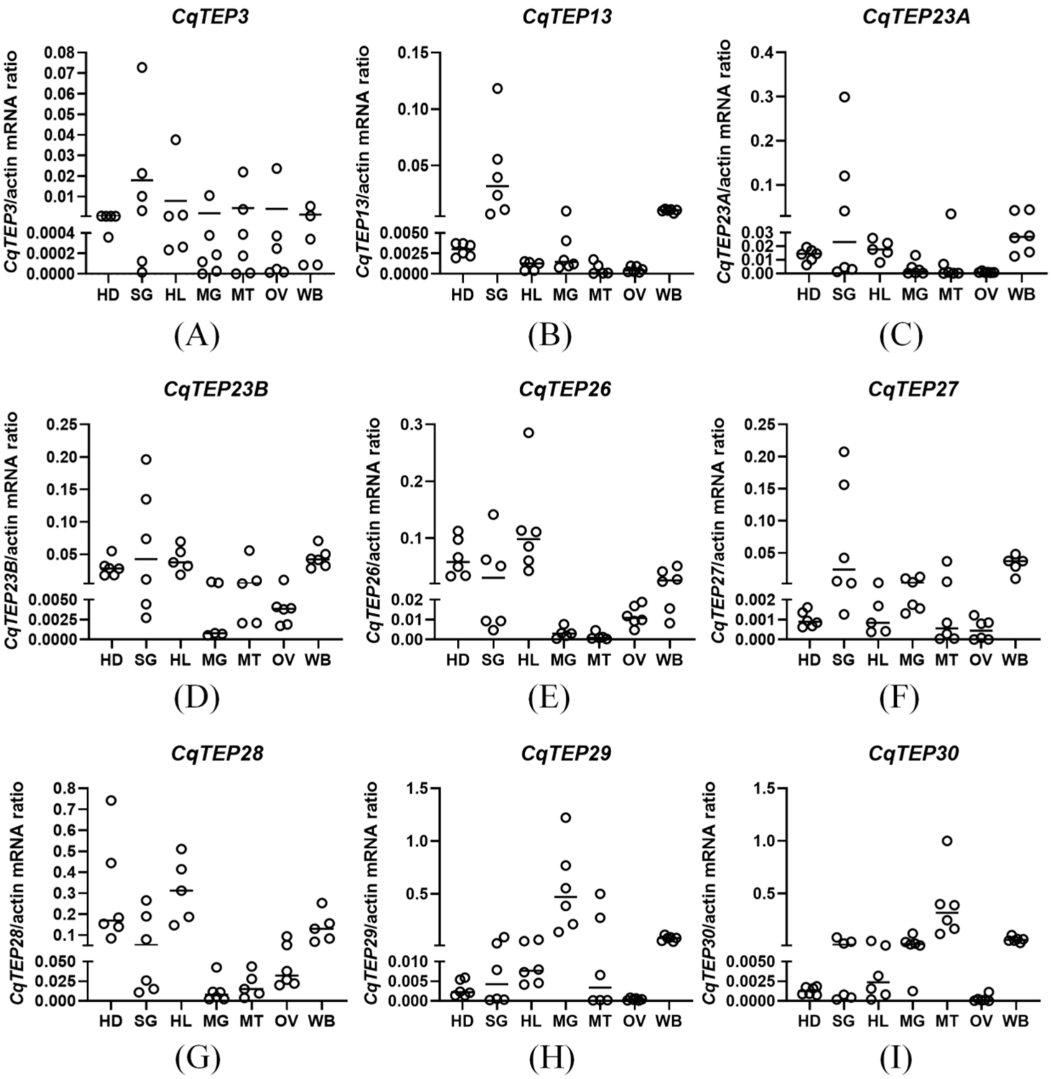

Figure S3. Expression profiles of thioester-containing proteins (TEPs) in different tissues of female *Culex quinquefasciatus*.

(**A–I**) Relative mRNA levels of *Cq*TEP genes in *C. quinquefasciatus* in head, salivary glands, hemolymph, midgut, malpighian tubule, ovary, and wohle mosquito quantified via RT-*qPC*R (n = 6). The results are expressed as mean. Abbreviations: HD, head; SG, salivary gland; HL, hemolymph; MG, midgut; MT, malpighian tubule; OV, ovary; WB, whole body.

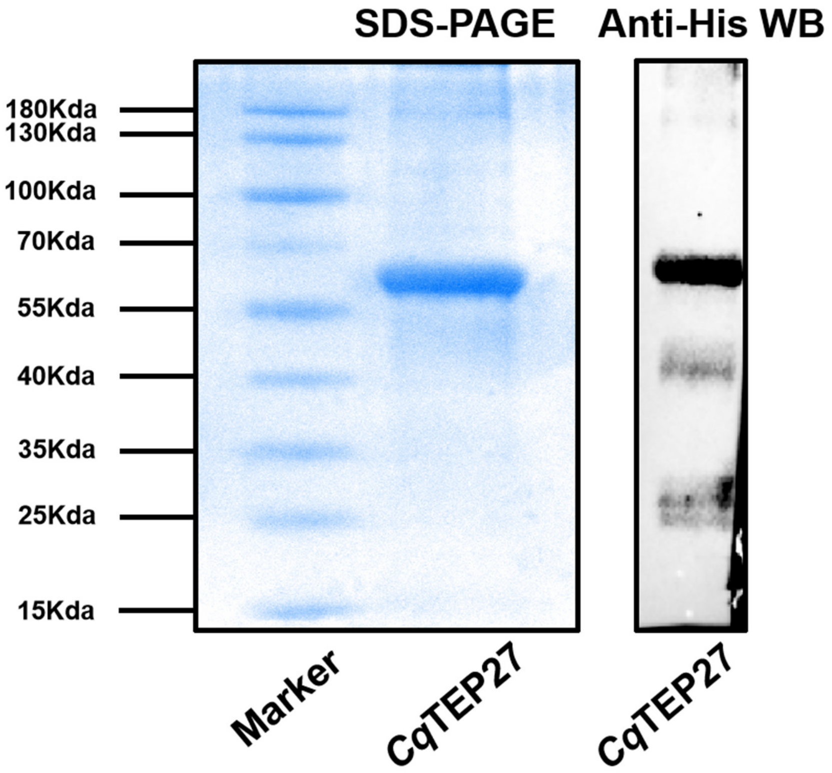

Figure S4. Purification of recombinant *Cq*TEP27 expressed in *E. coli.*

Left panel: SDS-PAGE of purified His-tagged *Cq*TEP27 (pET-28a(+)); right panel: Western blot probed with anti-His antibody.

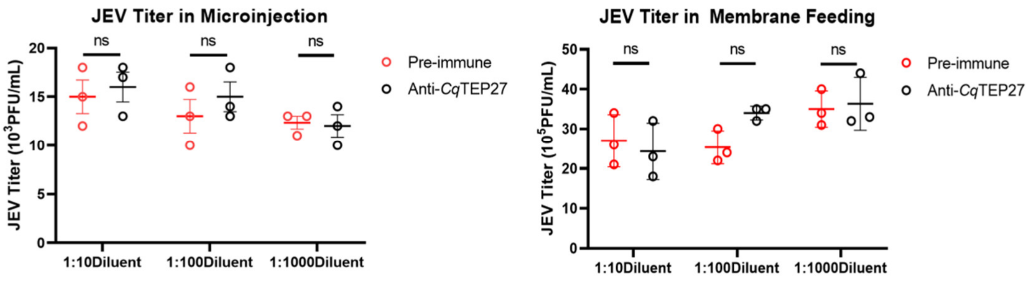

(A) (B)

Figure S5. Anti-*Cq*TEP27 antisera exerts no direct effect on JEV titer.

Plaque assay assessing the direct neutralizing activity of anti-*Cq*TEP27 antisera against JEV. JEV was incubated with anti-*Cq*TEP27 antisera or pre-immune sera under the same conditions and dilutions used for (**A**) microinjection and (**B**) membrane feeding ($n = 3$). The resulting mixtures were then subjected to plaque assay. Statistical significance is indicated as follows: ns, not significant.

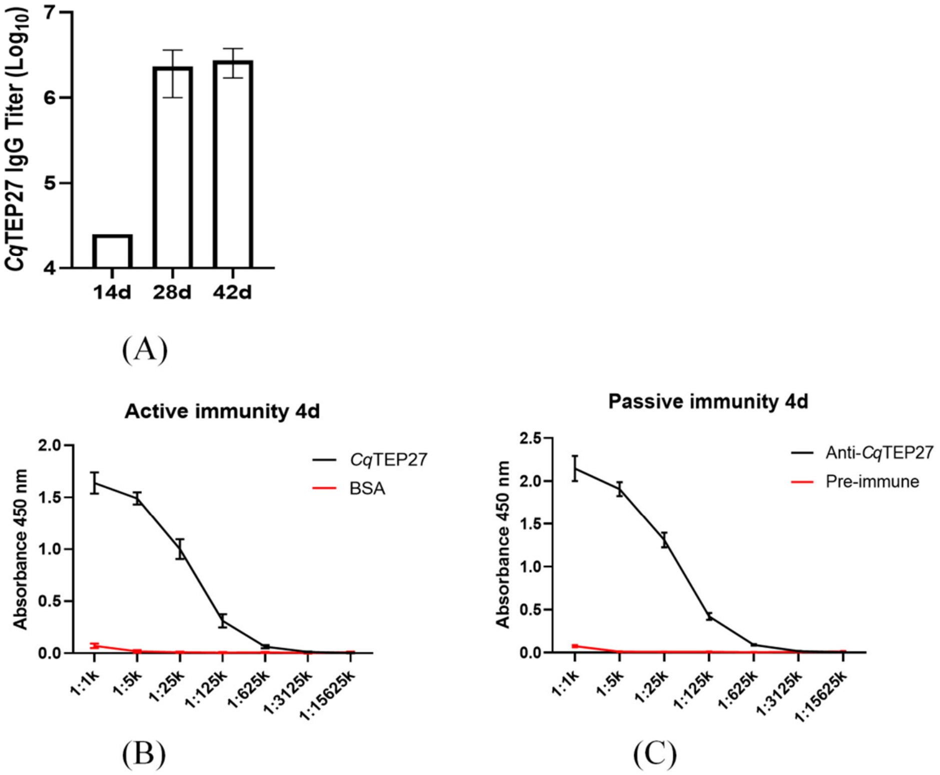

Figure S6. Sera antibody titers in A129 mice.

(**A**) Serum antibody levels following initial immunization and two booster immunization with recombinant *Cq*TEP27 protein (n = 6). (**B**,**C**) Serum antibody titers in mice at 4 days post-infection (dpi) under different immunization regimens. (**B**) Passive immunization group, achieving a titer of 1:625,000 (n = 5). (**C**) Active immunization group, achieving a titer of 1:3,125,000 (n = 6). The data are presented as the mean ± SEM. Abbreviations: **k**, one thousand (1000).

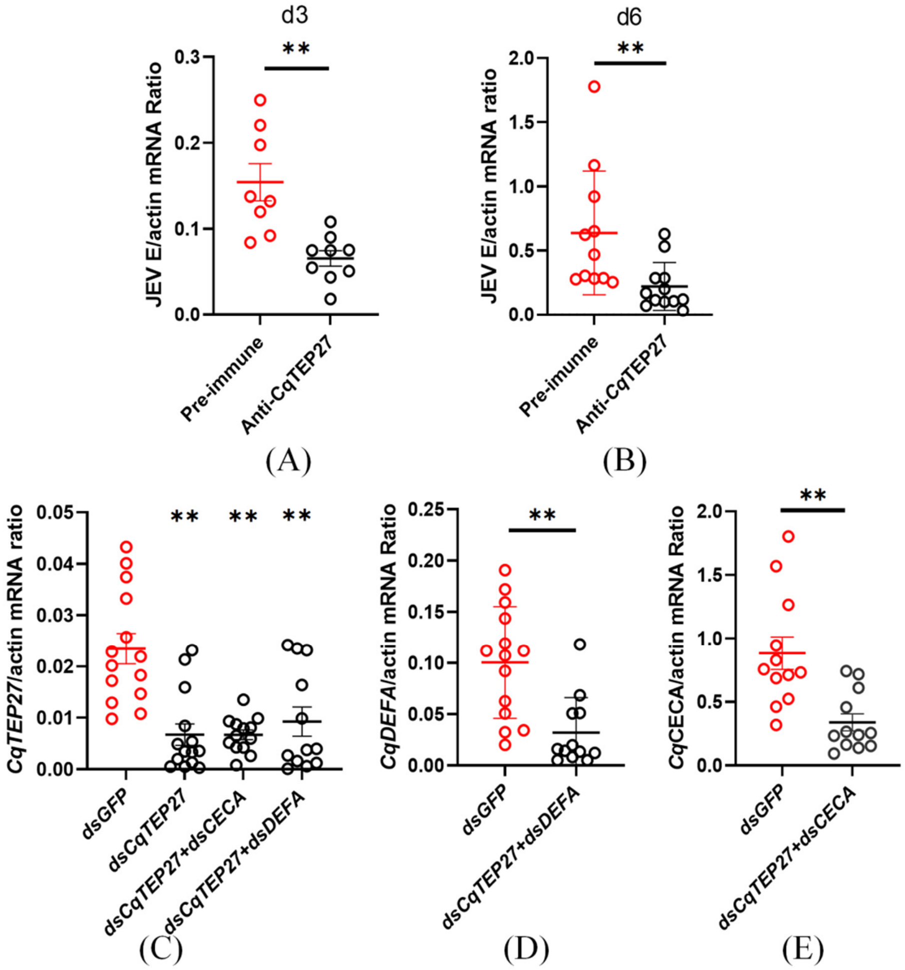

Figure S7. JEV infection load and related genes knockdown efficiency in *Culex quinquefasciatus*, related to Figure 5.

(**A**,**B**) JEV viral load in mosquitoes at (**A**) 3 days ($n = 9$) and (**B**) 6 days ($n = 12$) after injection with anti-*Cq*TEP27 antiserum in RNA-seq samples. (**C–E**) Knockdown efficiency of *CqTEP27* (**C**), *CqDEFA* (**D**), and *CqCECA* (**E**) in samples from Figure 5E ($n = 14$). Statistical significance is indicated as follows: ** $p < 0.01$; * $p < 0.05$; ns, not significant.

Table S1. Primers and probe used for qPCR and genes cloning.

**Cloning primers**	**Upper primer**	**Lower primer**
pet28A-*Cq*TEP27	ACAGCAAATGGGTCGGGATCCCATGTTTCATAGCGATTCG	TCTCAGTGGTGGTGGTGGTGGTGCTCGAGACAAGAGAAAATATCACAATCT
**Primers for SYBER GREEN RT-qPCR**	**Upper primer**	**Lower primer**
*Cq*TEP3	CCGTTCCGGATCGCACTCTT	TCGCTGACAAGTTCACCATCG
*Cq*TEP13	TGTACGATTACTACGCTCCT	CACCTCGCAGATGTTCAG
*Cq*TEP23A	CATCCTGACCGAAGATCCCG	TCTCCCCACGGGCTATTACA
*Cq*TEP23B	CATGCGCCAGGAGAAAAGTT	TGGTCAATCGCCACTATCCC
*Cq*TEP26	TGTCACGGATGGCGAATACC	AACACTCTGGGATAT
*Cq*TEP27	ACGTGCTTCGAGTGATTCCA	CAATTCCGACAGATCCGTTG
*Cq*TEP28	TCGAAGGACGAACTTCGGTG	CACGCTAACCCTAGCCCAAA
*Cq*TEP29	TGCTGGACAAACCGGTCTAC	AACGGCCATCGCCTAATTGA
CqTEP30	TCCGTAGACAGTAGAAGGC	CGATGGTCATTGAGGTGC
*Cq*actin	TGCGTGACATCAAGGAGAA	GTGTTGGCGTACAGGTCCTT
*Cq*CECA	AAGGGAAAGTCAACCAGCCT	AGGACGATGACGAACAGCTT
*Cq*CECA2	ACCCTTCAAGAAATCACCATGAAC	GGTTTGTCCGAGAAAGGCCA
*Cq*CECB	TCCTTCAACGATCACCAACG	CCGGAATGTCCTCCGAAAAC
*Cq*DEFA	GCAACTCTAAGAAGGTCTGCG	CACTTGAGGCTGCTCACTTA
*Cq*DEFC-like	ACATCAGTTAGCGGGGATCAA	AGCAAACCAGGACGAAGCAA
Mus musculus GAPDH-1	TGCTGAGTATGTCGTGGAGTC	GGTTCACACCCATCACAAAC
**Primers for double-stand RNA synthesis**	**Upper primer**	**Lower primer**
dsGFP	TAATACGACTCACTATAGGGGTGAGCAAGGGCGAGGAG	TAATACGACTCACTATAGGGGATATAGACGTTGTGGCTGTT
ds*Cq*TEP3	TAATACGACTCACTATAGGGAATCGCATTGACCGATTAC	TTAATACGACTCACTATAGGGTCCAACGACCGTATCTT
ds*Cq*TEP13	TAATACGACTCACTATAGGGCGTACATCCTGAGCAAGCG	TAATACGACTCACTATAGGGTTGGGATTCACTATTCGGT
ds*Cq*TEP23A	TAATACGACTCACTATAGGGCTCGCCTTCTCGGAACAACT	TAATACGACTCACTATAGGGGACGTATCCACTGGGCAACA
ds*Cq*TEP23B	TAATACGACTCACTATAGGGCTGGCACCAAGGGATTCAA	TAATACGACTCACTATAGGGGCGTTGAGATACGAGCCAC
ds*Cq*TEP26	TAATACGACTCACTATAGGGAAACAGGCTATCATAATCAG	TAATACGACTCACTATAGGGGCTGTCAACGCCAAGTC

<table>
<tr><td>dsCqTEP27</td><td>TAATACGACTCACTATAGGGGCCAGTTATGCACTGTTAGG</td><td colspan="2">TAATACGACTCACTATAGGGAATACATTTACCGCCAGAT</td></tr>
<tr><td>dsCqTEP27-2</td><td>TAATACGACTCACTATAGGGCGGTAAATGTATTGAAATCCAC</td><td colspan="2">TAATACGACTCACTATAGGGACAAGAGAAAATATCACAATCTTCA</td></tr>
<tr><td>dsCqTEP28</td><td>TAATACGACTCACTATAGGGACTACAGGCAGCAACTCCAC</td><td colspan="2">TAATACGACTCACTATAGGGTCGTCTTCGCACACATCACA</td></tr>
<tr><td>dsCqTEP29</td><td>TAATACGACTCACTATAGGGCTATCGTTCGCTTGGTGTA</td><td colspan="2">TAATACGACTCACTATAGGGTAGTAGGTGCTGTTGGGTA</td></tr>
<tr><td>dsCqTEP30</td><td>TAATACGACTCACTATAGGGCGAGGGTTCGGTGTTTATC</td><td colspan="2">TAATACGACTCACTATAGGGGCCAGTTATGCACTGTTAGG</td></tr>
<tr><th>Primers for Taqman RT-qPCR</th><th>Upper primer</th><th>Lower primer</th><th>Probe</th></tr>
<tr><td>JEV Envelope gene</td><td>CTGGTCCATAGGGAGTGGTTTC</td><td>CTCCACGCTGTGCTCGAA</td><td>FAM-TGACCTCGCTCTCCCCTGGACG-TAMRA</td></tr>
</table>

Table S2. The protein sequences used for phylogenetic tree construction.

Name/ Gene ID	Amino Acid Sequences
AaTEP22/ AAEL000087	MIMNSVIVLVCSFIVVAKCQSSSVLVIGPKYIRPRHPFNVAFANSLNSNVNLKLTLQCDGDNGFVKNASLRLNYQSAKSFSVDVPNITSGTDCTFSAVNDGGSVM<br>VDHMANLLLPAKTLSVFIITDKPVYKPGDILRFRVVVVDIATKPVKHMESIVIEIIDSDGAFKREWLQARLLNGVFEAAYRLPSVPALGIWNITATANDNGFENTK<br>VQDFEVREYVIPKVVLKVVPSRTLLIAEKEISLDVKAHYTFGEPVEGKLRVDLFTNPLFRRATHSVEKSFNTNVQIKFKLDRELALRDANFCMVSANVSLTEKLSN<br>VTTVVTLEFPVFRHPYKIELINPAMNYRPGVGYTCKLSVKDHFGIPVDAQGNSITVQSDTDSVTGQLDRQGMVTLTLPMPDSEETVLISVVYENVEYNKIIEVEAS<br>EDLSTQYLHISTKTRIIVGNSITFTVNSNQHFTHMSYFVTNWGGILLAGHQKFSRKKTNTIRFKLTAAMSPYSRLLVYTISGGQLIMDYFELDFEFFGNEFEFMLDD<br>ANYRPDQDIYVDVKAEKNSYLAFQAIDQGALLLGFDEFGLTRKQVQEDLASYVDADEENRLDLIHSFGLFLRSGFNETKTQSSRAKRFAPERRDRPYRNAIRLRT<br>DFSESWFWKNSTMKNQKTQTFHDVVPDSITSWYVTGFALSPTLGLGLMHAPRKFTVTKPFYMVANLPYSIKRGEVVRIQIMLFNFLNSDLTTDVTLFNKNDEIDF<br>VDRLSNNPHFRTKAIIAPHNQVKSVSFLIKAKKLGEIAIKIEAVNQLESDGLEHMLRVTPESRLYEKTEARFIDLPTRNKTNFPITCNIPRDADPGSTKIEVFIDPPLM<br>GFLAQNTTDSLLKIPTGASNLNLLTFVPNIVLLEYLKETGKVTPSIEQARNYVSSGYKNQLKYKHSNGAFGQWNPPRGNPSVFLTALVANALATASKHIDIDKKIV<br>EQAYSWISGKQKPNGCFEDDGEVIYTPLQNNSSSFALTAFIVSAIMENENIARQFAAVVQKATNCLAGNFDSLNNLHDIALTTYALALVRHEKRQIYLDRLIRDSIF<br>EKGSSTERYWNEELNVEIAAYALLSYLQIGNVIDTMPIMTWLNKQRYSTGVFSGVQRTFVALKALGKMATYLNTSKNDYSVYISYDKHKSKFDVLSTKSLETFH<br>HELPDSVRNVNFAVEGIGFGYLQLTYQYHRNIQNAKASFMLDVTVLDSSNYNVQDLRVCLSYKPKEKYTVSGVALVEVYLPSGLIVKESAVRDISRQIQRTERAF<br>DNTAMFVYFNGLDTNSVCFDITAHRKFKIAMHRPSYVVVYDTNDMSGFAIKSYEGKVLQICDICDDEDCRSMSC
AaTEP23/ AAEL001163	MCPLLIATLYFILQYTAPANSSEVGHYIILVPTVIHDHSKVSIGLSTVGYEENKQFQVILKKEQYDDKEGEVVNEKHRQSSDTRLVQFTVSSLDNEPYQFLIHDPDG<br>SVQTTHVEVSKRPVFIFIQTDKPLYKPGDTVKFRVLVLNHLTRPIDSLKVINVQLKDSNSDLIRQWHYARLQKGVFQSQIDLANFVPLGNWTLKVSALNGIEETKT<br>FNVAEYVLPLHEIHITASKQVQMNDEILKLIIDAKYTFGKPIIGYVTLAFNELNYPDMYEINGRAVISIPLSDIVDTDVGDDVYHLNVQVKIEEDESEQIYEASETIPI<br>HKRSYKITLRKSSEYLLEEESIWCWLTITNPDGMPLTNRNVMTVKVTEMLGSRSNKHYSFQKEPDDEGVVSLKIDSSNTTDRLELEVTYEGETTKFEILHGSDNNN<br>TRNDFIKASLLVQKPMLKHPVEVLVQSSFRMNLVIYYVISQGEILASGRISVNFKRTATFSYLATFPMVPEASLVVFTINDAMLWKDIVRFKVHELDNFVDIEISSN<br>SAEPRAQIFLEVKSNPGSTIGLLAVDRSLLQLGTGNDITQQIVLEKLGKPKIDEDVELERLGFTVLTNAKSHFMASPLGSSPHLLTQRFGDDEELHLGQDMQADHG<br>TRILRKNFPETWLWTEMIEVDDKGHVDITDIIPDTMTSWSISAFAINTNHGLGVVKNPVALTVLKPFFVTVNLPYSIVKTEQAVVEVFVHNYLNQAQHVTVRVQN<br>AQKDFIHSDLEVSKTVFAPSNSVKTVTFALKPKRSGNLTVTILADCSLATDAIQRNLRVTPGGLQYFENSARFIEVQNSSMSFDPIKLVIPRTATYGSVSITFSVEGF<br>LLGAALTNLDHIIRLPSGCGEQNMLNLVPSVIALEYMDNTDTLTYGIKAKAIDYLQKGYQNQLKYKLRDGSFSVFGQSDGRGSVFLTALVAKVFTFAKRHITIDN<br>YVIEKAFNWLRERQLSDGRFVESGRIYYKELQSGVQDGTTLTAYTLIAFLEHKSLTQKYLSVVNKGTEYIAKTYRFDGNPYSLALIAYVLQLAGHPRKHYFFDKL<br>VELSKINEDRTMRWWGSGSTSIETTAYVLLTYMSRGSYIDAKSIMRWMVSQRYDKGGYSNTQNTFVGLQALGKYSRVMSLSNQNYDVFVNYDSERQRLHMNS<br>TTSLVGHKFNIPSDVRQVKVDVEGTGAGVFQIAHKYNNIAHDSVPRFKIEKTLFKIRSDGVANMTIQAIYRPKKDFEETNMVMIEVMFPNGYVVTNSQLKQLEKN<br>NQIRRSETADGDTRLILYLDPMRPNNPVYVDVEAFRKSTVLNQAPGWIKVYDYYDPTREAIEYFDPISN
AaTEP20/ AAEL001794	MGMEKRSRTVPARRRRRWSIQANMWILPLLICCGMLIERVQSQGFSVIGPKTIRPYSTYSVAFSNSVNKNVHLNVILEDQNNPSEFSLKKLAVVNRRTVKKIDFDIGNIT<br>SSEYQLLVQSADRTFSFDQRVELLYEPKTMSVFIQTDKPVYTPGDLLRFRVIVVDADTRPVTSIKTVNIAIDDSAENSIRKWPYAKLLNGVFESQVQLASSPVLGTWSIT<br>VKASDDIIVTKQIEVKEYVLPKFFVKVYPSEVLLVQNEKVSLTLEAYYTFKEPVDGNYKVELFLDHTKRKPDFIKSDRITGKTSLEFQLKNEVDIDGDEQYTDVTVKVE<br>VVETFSNRTVSITEKIPIYRQPYAVTLLPSAPSFRPGVPFDVKIFVKDQLGHPPPEENTASIDLTVEFHLPTDSDTKSLTVDLDEKGTGQFILLPHPNAQELKVTATYDSQE<br>YEVIHDPIHGFSSQSKQYIKVTLNPKYYNNIKVDKDIVLDISCTETMTHFSYIVVTKGNIVEASNVPVAKKKKHSLRLKMTSKMSPESRLLVYYTNREYLIFDDIELKFD<br>SFNNDFKFDLNDDEYFPGQSVYIDVYASKDSYVAFSGIDESVLLVGKERHDFNKGDVLKELALYGATNDAEFDLFHKYGLFLKSTATVDTPVTRSQNARFGTLLGRT<br>KQAIEIRTQFLESWLWKSFSMDGRNNFKAIEDSVPDTITTYHVSGFALSPTLGLGVIQQPVSFTVRKKFYLVANLPYSIKRGEVALIQVTVFNFLGSSVTTDVTLFNKRD<br>EIEFVEKASTNNTHRTKAVIAPNNNGKPVSFMVKAKKLGQIAIKFQAENLLETDALEHMLRVTPESHRYEKNVARFIQLPTHSKVPFDVKLDIPKNIDEGSAQIKFTLDP<br>DILGTTISNLDGLIRKPSGCGEQNMLHFVPNIVVLDYLNETNTAAEDVRTKAINFLSSGYQNQLRYKRSDGAFSVWGQSHAGSTFLTAFVAKSFKIAAKYIQVDKSIVD<br>AAFDWLAKQQQSDGRFPEVGQVIHADMQGGLRNNGFALTAYVLIAFAENEEVYRKYRSQLIKTTSYIANNLDNMENPYDLSLSTYALMLANHGKRTEFLDKLVEISI<br>FDSNQTERYWDSKPVDIEVAGYALLSYVAAGDLLHATPIMRWLNKQRYGLGGFPGTQDTFVGLKALATFAAKVSSGRNDYRVTIIHEPNRRRTFDVDRHNAFNIQEL<br>DIPNNIRKMRVEVVGIGNGYFQVAYQYYQNIQVAKPSFSLTIDQLNTTTEHMQQLDVCVKYIPKEAYQKSNMALVEIFLPSGLVADSDAITDKTGGIRRIERRFSDTSV<br>VIYYDNLGPEDECFRVTAYRRYKIALHLPSYIIVYDYYNSERFAIQQYEGKVLQLCDICEDEDCETLSCENSSK
AaTEP3/ AAEL008607	MGHLTMSPPWALSLMVLISVFVVSCKCEGYYTVVGSKLLRPNSEYHVSVSNLNVREPLRFRITLNNTRTGTPLASEDLSLGQGESRLVPFSIGDITEGDYSLTAEG<br>LSGFIFKNESSLTFQSKSFSVFVQTDKAVYKPGDTVRFRVLVLDPNTKPLQKIDTVKVHITDGKNNRIKQWNDAKLVKGVFESELALSSAPVLGNWKINVEALGT<br>KKTQEFEVDEYVLPKFEVSVESPGITTLKDGKVKAIVRAKYTYGKSVKGEATVSAYPDFNFHYVQPFERDVITRKTVPIDGKGSVEFDLRDEIKLQGDYTRNIVIE<br>AVVEEELTGRKQNSSSKVKIYDRAYNMELVKSSEKFKPGLPFTAWLKATFQDGAPLQDDTNSVKVTQEFGWPNQNTTEHTYKLDKNGMAKLIIDTETSSDSISF<br>RAEYLGATFYLGSISQGWVKYSAYVRAKVLTETPTINKDVTVDVSSTVPMKYFNYQVLGRGDVLIGGTIAVPDRMSHTFRFPASFAMVPRARLLVYFIHSDGQM<br>ISDFAEIEFASELQNFLKVQLSKTESKPGQDVDITISTNPDSYVGLLGVDQSVLLLKSGNDLTTGKVFDDLKTYEEAVYLQYRRKRFAPWRRFNYYDDFNDAGAV<br>IMTNANEQPAIPPWASYASFDGGVIDIRTYENEDPFAVPLAASAGVMPPSGVGGAELSVRKSFPESWIWQTFSNDSFSGEKTITKKVPDTITSWMITGFSVSPVYGL<br>GLTRQPRKLNVFLPFFVSTNLPYSVKRGEVVSIPIVVFNYMDSDQTAEVTFHNTEQEFEFADVENEVHENPKLELFRKKTVQVASNTGKTIPFMIKPSKLGHITIKV<br>TATTQLAGDGVERQLLVEPEGLPQYVNKASFVDLRATPEVNNNFTVEIPKNAVPDSTRIEIAVIGDVLGSTIQNLDSLIRMPYGCGEQNMLNFVPNIVVLDYLKNT<br>NQLTASIEDKAKKYMESGYQRELTYMHDDGSFSAFGKSDPKGSTWLTAFVARSFKQAADHISVDEKIIDKSLEWLSDQQSSNGSFPEVGKVSHTDMQGGSGQGI<br>ALTAYTLIAFLENKNLIPKYTNVINKALDYIVRNTEGLDDNYALAIAAYALQLADHSAKDFTLSQLDTKATTDEDQKWWNKPIPEADKKNPWYSKPNSVNVEMS<br>AYGMLAFMEAGLDSDALPIMKWLISQRNDKGGFQSTQDTVVGLQALAKLAAKISSKNNDVTIVVTYNENQQKEMKINSENNMILQKFELPSSAKDIDIKATGRG<br>FAVVSLGYKYNMNVTGEWPRFVLDPQVNKNSNQDYLHLTVCTNFVPTAGQNKSNMAVMEVGFPSGFTADSDTLPSLENTEYIKKVELKDGDTVVVMYFDSLD<br>RNELCPTVSAFRTHKVAKQKPAPVVIYDYYDNSRIARQFYNGPAAGLCDICENEDCGNSCSIKSQKQRSPKEEATVMAKSGAVGITIGLQLIVAALLVRLFN
AaTEP1/Aa MCR/ AAEL012267	MTRRWHPTWSGVLLLLFGFPSILTWAQQQQNPNDVPRYNPLDPFNRQRYNPTSSNFQPQSNPTASNDPRNPSFGSRDFYGTDSNSIDNRFDPSRGDRDRDRDVLF<br>QSTTPREFPGTRFGTTPRPRTFNPQSNRNVFLDQSNNPLNKEITYFVVASRMVRPGQIYKVSVNLLEAQHPMAVRASISRDGVELSSELKSVRVGIPETLLMRIPPTS<br>VVGDYKLRVEGSYENTFGGYIFVNETKLTFSQRSMTIFIQTDKPVYMQGEMVRFRTIPITTELKGFDNAVDVYMLDPTGHIMRRWLSRQSNLGSVSLEYKLSDQP<br>MFGEWRIRVIAQGQIEEGKFSVEEYYQTRFEVNVTMPAFFFQTDPFIHGKIMANFTNGTPTRGNLTLKATIRPIGWMNPKAINHMNRVWNTGNRRELDPNIPYYL<br>QYTNPDLFNAQSTFTSQLNQPDSQYQDQRYQSNYQDSYVIERHFNFDEEWPFWIKKPIDSAEQWDSWTNTYRESLPYLRYFNGTYHFKFPMSELSQLFQSNLAG<br>MEVLITARVGERFYDEVIEGYAMTRIYNSSIRVAFMGNSPQVFKPSMPFTVYLIAEYHDGSPLPIDPLYPGRMELSGTIDSRSGGGRNTYDVKELQMSDKPGVWE<br>LKIDLRNDLNLENSKQTNEFLNQIQSMRLSANYIHPSGERASAELLLLSHFSPNNHNIKVLTSTRDAKVGEYMIFHVQSNFYIKDFHYIVMSKGIVLVTGQEIISGG<br>VRTLSITLSAEMAPTATIVVWHIGRYGKVIADSLTFPVNGISRNNFTVFINNRKARTGEKVEVAIYGEAGSYVGLSGIDNAFYTMQAGNELTYANVITKMANFDE<br>QTNGTFKQTWISHEGDPDELVYYPSSTFGIDANRTFEYVGLVVFTDGVIPRRPLSCDPAMNYSECLSGRCYRSDKRCDGYFDCEDGTDEAGCDNRNSTALAEFRK<br>YRFNRILRHYQNVWLWKDINIGPHGRFIFNLEVPKIPALWIVSAFGVSTSQGFGMLRKPLEYVGVQPFFINLEMPTVCRQGEQVGIRVAVFNYQTVDIEVTVVLHS<br>SPDYQFVHVEEDGIVRSYNPRTSFGEHQFYIYIYAQDSSNVYIPIVPTRLGDIEVTIHASTLLGAYQVSRKINVEPDGLVQHRHQSTLLDLSNRAYFFQYMHVNVTE<br>TPIIPYEIDRYYVFGSNKARISVVGDVVGAIFPTMPVNATSLLSLPMDSAEQNMFSFAANFYTIQYMRAIKQRNKKTEKLAFHYMNIGYQKQLSYLNDDGSFSLFR<br>ADWNQSASSVWLTAYCARIFSEASFYEYENFIYIDPLVIQKNIHYILQHQKDDGSFWEVTWLPDRKVNQSSEHPRNEIVRSKNITLTAHVLITLVSVKDLSGRLGSS<br>VALAEQRASRYIERNIGLIKDYGSPYEIAVVSYALAQAKAPQAEHAFKILASKMRSIADLNYWGNDEVPQPPSKLENQKYFNLPRLPYKYDSVNIETTAYALLTY<br>VSRQETFTVEPIVRWLNSQRLMDGGWASSQDTGIAMKALTEYSTRNRVADVTSLTVTVEATSLPGESKVLHIGQHNLATIQDIEIPNAWGTVKVQAKGVGYAIL<br>QMHVEYSVDTYKFQTMPPVKSFDLTTHTVFHGRNQSHISYVICQRWTNTAESIRSGMAVLDVAVPTGYMIQQQKLDSYTISQRVRNLQRARYQERKVLFYFDYL<br>DNDFVCVNFTLERWMPVANMSRYLPIRVYDYYAPERFNETIFDSLQTYLLNICEVCGSSQCPYCRIYNTAIRNPVSVLLLLFTSVLIVARHYRIVNPNSWIFWND
AaTEP24/ AAEL017023	MVKYGECTFLCALLSVVYTQSQTTGNYVILAPKYLRAKHPYQLSIATHDFNGFTKLHLAIDGYTENDEVANVIKEVHLKRSQTQLVEIDTTTLPPGSFSLNILSGS<br>GGEVNLTIPLTVLDKAYTVLIQTNKPIYKPGNVIKFRVLLLDEATKPVHKPKAIHVTLADPDGNEIKVWPYAMLQNGVFQSQLEISNEPNLGNWSITANAYGKDH<br>TFSFLVDDYKLPKYELKVSTPQPATVSDREFSVDVEARYIFGRPVKGNATITVNGPKKQSKTAKINGKIRLSFSMIDLLHQTSIVDEIIPVEVIVVIHDQYTRENIKAT<br>NMFRIFNQSYRLSLKKSSKYFIPGHPYRCIVEIKDQNGRRLTSIENHQALVNVVYSGPNGYHNTSEMRLNPESDGTVPLTLEVPEQVTHIELNISYRDTNEHFELQRI<br>HFSTTGQIQASIITEQLTLNTPIMVQVQSSIALNHLTYQIIAKGKIQTVQQMRFDDTDSVQFNITATPEMIPNAKVFVFSMHNGLILKDTVPLMISSLPNWVNVTLPN<br>EKVQPGSRIQLEVESTPNSLVGLLAVDRGAWLLGDGNQITKQSVLDEIGTFSDEIEGDNDDISEMELITNGIIPTALTARFGNFVPDEEDAYIPPRKEFPESWLWLDL<br>QKTGMDGKLHIADIVPDSITNWEITAFSISPEHGLGVQDEPVALAVSKPFFITINVPNSIKKSEVAIVKVAIFSVLNDTSYVGVTLKNSRQEFEFVDNRGRKDVSYQ<br>AKNVIVTANSATTVLFKIKPKKMGNIVIKVIAETTEASDSTEQLLRVTPESLPYSITEKRLIQLQNNRQSFELELKIPRHIDVNSEEIHFSVQGNLLGDSVDGLDEMIR<br>MPTGSGEQNVLKMVPNLVLLDYMVGVGKVNVPLRNRAIKFLGMGYQNQLKFKRNDGSFSMFGQQDNAGSVFLTALVAKTLHQASQFITVDEKVIENAYDWL<br>RQQQKMDGSFTELGNIPEYGLQRKHTEKSVLTAYTLVAFLENDRISEKHKSIIDKGTQYLVSKIQGLESSYALALTAYALQLAQYKQEYAFSKLLENSQSNNGFR<br>WWNDDNTAEATAYALLCYIQRGDFVDPLPILRWLISKRHLFGPDNIETTFIGLQAIAEHSKRISPNRNNYEVSISRESNQLVTLNINPETSLTVQNVSLPSNVRKVR<br>VMVNGTGTGTFSIHYRYKTNILNLRPRFDVKVQTLDTTTSHYLDLKICAKFKPSEAYEISKLALMEITFPSGYIALDESVEELEKMDTIRKITTKYDDASLWLYFES<br>LPEHFLCIPVTSFRQSDVLQQIPGSVRVYDFFDDSRVAITHFDGKQLDKCEICDNDCLPECGN
AgTEP15/ AGAP00836 4	MRRSTMFASKKDPAIRILSTSSSSLLAVCLVLSVALVPAVQCEGHYSIVGAKLLRPNSEYHVAVTNQDVSEPIRFSLAITDASSVIAKQEITLNTGETRLVPFAIGDIS<br>ESSYKLVAEGLSGLTFKNETDLEYQQKSFSVFVQTDKSIYKPGDTVRFRVLVLDPNTKPLQKADNISVHINDAKANRIKQWKEGKLVKGVFESELTLSTAPVLGA<br>WTINVEVLGSKHNKVFEVDEYVLPKFEVTVESPGITTFKDGKVKAIIRSKYTYGKPVKGEATVSVSPEFQFHYVQPFAKDVITRKVIPIDGKGSVEFDLREDIHLEG<br>DYSRNIVIEAVVEEELTGRKQNASAKVMIYDRRYKMELVKSDDNFKPGLPYTAWLKVSYQDGAPVQDQTNPVEVKQSSFESTTSVQNYTLDQNGMAKLEINTE<br>VNSSYINVVGVYLGQEFYLHGISKAESDVDSYIRAQVLTEMPLVGKDVLVEVTSTSPMKYFTYQLLGRGDVLLSNTIAVPESKTQSFKFPATFAMVPRAKLVVYY<br>IAPNGDMVSDSKVITFDSELQNFMKVSLSKEQSKPGQDVEISISTNPDSYVGLLGVDQSVLLLKSGNDITKQQVFSELEKYEERSYGFYRRKKRFAWNPHAEHRD<br>FSTVGAFVMSNANDPPPNNTLPEVPIYKFHRAPNGTVLYTTIEKPRAQKQHHVLVTNTRPPLAGPFAFSRIPRPHRDIPRLFLSQEIQNTWLFDNTYSGFSGEKTLQ<br>KKVPDTITSWIITGFSVNPIYGLGLTQQPRKLNVFLPFFVSTNLPYSVKRGEVVAIPIVVFNYMEDDQTAEVVLHNDEQEFEFADVENEVVESNKVELFRQKRLDIA<br>SNTGKSVSFMVKPKKLGHITIKVTAKTKIAGDAVERQLLVEPEGLPQFINKAAFIDLRAAPELTKTFEVEIPKNAVPDSTRIEVAVIGDVMGSTIQNLDSLIRMPYG<br>CGEQNMLNFVPNIVVLDYLKATNKLTANIEAKAKKFMEAGYQRELGYKHRDGSFSAFGENDKSGSTWLTAFVARSFKQAANHITIDEGVIDKSLEWLSDHQAP<br>NGSFPEVGVVSHKDMQGGSGSGVALTAYTLIAFLENINLVDKYKNTINKAIDYVYRNTESLDDTYALALAAYALQLADHSSKGLILSKLDTKATTDSDSKWWH<br>KPIPETEQKNPWYSRPNSVNVEMSAYGMLAFLEAGLDTDALPIMKWLIGQRNDKGGFQSTQDTVVGLQALAKLAAKITSPNNDVTLTAKINENQEKRMTVNAE<br>NGMILQKFELPSAARNIEIQATGSGFAVVQLSYKYNMNVTGEWPRFVLDPQVNANTNPDYLHLSVCASFVPSAGQNVSNMAVMEVGFPSGFTADSDTLPSLEN

	MPFIKKVETKDGDTTVVLYFDSLDQRELCPTISAFRTHKVAKQKPAPVVIYDYYDNSRIARQFYDGPKASLCDICENEDCGEACSIRSQKQRSSDSPSRQPTVEGT<br>MQSGSQTVSVSFFTFLLATLLVRMFH
AgTEP2/<br>AGAP00836<br>6	MFSKGGGMRFGGEVKRTVPDPKDHKDGHYSIIGARILRPNSVYRCVVSTFDTKSAIVFRISIAAKDKPIATEEITLNSNESRLISFTIDSIPEEEYELVAEGLSGLEFKT<br>KSRLDFDNKFCSVLIQTDKSVYKPGDTVRYRVLVLDRSMKLLPAGDSGMMVYIRDGKGNRIKQWSNASLGECGVFQAELTLSTEPVLGEWTINVEVVGLKESK<br>TFDVDEYVLPTYEVTVESPGYTFLDDELLKVVVNSKYTYGKPVAGELTVSVKLASSMCFRREPTETSICQKVLPIDGKTDVEFNLKEILSSKTYIRELTIEAEVCET<br>LTGRTQKGSTTVQLHDERYQVRMIEESSYFPGLPYNAWIQVTNLDGSPVQDGAKEVEIVLRNYNIDLHKQSSTLDDKGMAQLNVKLDELDFDYVSVEVKYRGK<br>DYYVQGITKPRDYEEALMRVRLSEKEPTAGKDLTFDVACTKPLQCVSYSLLARGELLAGGAVKGSEASTTISITIPSTFAMVPRAKLLVHYISSAGYIVSSYDTVEF<br>KRVFENQIQLTLSKDELKPVETLDIDIRTEKDSFVGLLAVDQSVLLLKSGNDISRDEVVQQLEMYESAQNYHWDAYSTSDCQSVGAVLLSNRFIPRDIFPQARLFA<br>CSTSAGGFGAAPMMAACKMKGVIMESEMATAPVNEPTVRSKFPETWIWESISKCKEMESIRKIVPDTITSWIITGFSLSKSHGLGLVDNPSKVNVFMPFFLSIDLPY<br>SVKLGETIRIPVVVFNYMDEDQLADVIFYNNDDEFEFVSDTKDQKEKHRQEQITVPRGTGKTLTFVLKPTKVGHVTLKITAKCALAGDGIERQLLVEPEGLPQYIN<br>KALLVDLRLVKEIKQPFEVEIPVDAVPDSTNVEVSVIGDVLGSSIENLDSLIRMPFGCGEQNMLNFVPCIVVLDYLKACKRLTVEIESKAKRCMEIGYQRELTYKH<br>QDGSFSAFGESDKSGSTWLTAFVAKSFQQAAKHMTIEEDVIDSALGWLSKVQTADGAFPEVGTICHKDMQGGAGSGMALTAYTVIAFLENPKLGEKYKASVDK<br>ALTYVKEHISELDDVYAHALAAYALQIADHPLKNEVYASLLSKSNKQGDIQWWSKEIPEKNDSNCCWWYRPCSVNVEMSAYGLLATLEASSAGLEGLPIMKWL<br>VSQRNDKGGFESTQDTVVGLQALSKMAAQLSSSEADMSLKVIITGEQEKCLQVNGGNILVLQKHELAANTRKLEMIATGTGCALFQLSYKYNIKDVDNSPRFTL<br>KPEAKQGSIKSCIDLSITTSFIPKEDQAVSNMAVMEVDMPSGFIVESDTLKQLKQHEMVKKVETKRSDTTVVLYFDNIGEEAVHLQMSAFQKHEVENAKPANVII<br>YDYYDNTRCARSFYEIAV
AgTEP14/<br>AGAP00836<br>8	MASNAVFAMLLLVVLVVWGIPRDCAGQRYAMLAPRTVRPHTAYELMVSNLGSGTEQFMYEIVTANDTVVGATSVEVKPKELRKVEVSLNSLKEGHYQLKVW<br>EKQKQTLLNTTSLERIDRSYLVLFQTDKPAYKPGDRVQFRVLFLLPDTKPVGQSVRPTIFIADPDRVRMKQWNGVSLSSGVFEGSFQLAEDTSFGRWIITASVNEQI<br>YKDTFSVEEYTLPLYRVQVQSIPKAYFQCDEPKMSLKLSASFVHGGSVRGNATVVVRANYNNYPSQTKEVARKELFINGTAIVDFPTDVVAKSCDEERNVWFDV<br>IVTESSTGVSYNTTCSYTVHNAGGVTMEVLDGNEVFYPGLAMRLMVKLATIDEKPLVNQPVTVRYKVLDEDRNDIDEVAPSILSLQTNANGVFRVAINTTTSTAE<br>VTVEGVYRNHIFPLVFAYPMYEERSLEYLAITTRQAYCIVRRNITVDVHSNVKVDRIYYVGYCHGKVCASGVQKSATRVNTHHLTLTSSPQMAPQMKLLAFAVK<br>EDGKILSSSIIIRFLSSSSSALNVTEHLMEPNSDRYSFNILAEKNAFVGLLGVDERIKQRTSSNNDITQQKWEKMLQSLEGTLGTWSSYDSFGSVGVTLLTDGYLPD<br>VGFIPHSFGELARTTSTLDEDDSVREDFPETWLWESIKAKNGQTSIEKSLPDTITTWVVSGFSVGPANGLQILKKPLQIKSQKRIFVQLHMPPSIKRFEEVSVHCLVH<br>NYGKAINVSLEVRPTMKVTPLYLAQGATENVRVSVSSSSVGVLTLEVFLRDSKGKVLDSVRQSIPVRPEGLIRTVEDVRVLDFPQQSKLTFNLALPAIEQNLDVNY<br>REVTLSVVGTFLNLNMFDLEHMVRSSHGNGEETLLFLQTTMAVYDYLKRANLLSPDTKKKLFSYMDVGYQQLSRYRLDDGSFSMFGNLHECGGVWFTASTVQ<br>ALGKLIMYEAIPVEIDFLNDGLNWLILQSGEDGSFNESCPIAHPHIQRTGGKELSLASSVMFAFVGKEFSREYKQFINKTISLLLASPINDVYLLAKTTYLLTLINHPD<br>SARMLAKLNSLAIVDGKYRYWSVSGGDPRSSPEKRDQEATAYALLANIKRNAINHEEMIRIAQWLQKHTFAGDRSVASLERIVALEALAALAHKIPTTMPNMYIE<br>AGGHKFEINATNRELLQTVQLPQGTTGVRVSARGTGLVLMKLSVRYSLASKNATRTEPSNVSNSEKIGVNIKKRQVENHKLALYLCFTLLKPLLFYEPELWKVEV<br>DFPTGYEIEGQEEHYHNGTESAIVLENKSHLELVWNVRQVSQVCYPIVAIARWSFVDIVTGLLYLTSFDTKDTVAVYAF
AgTEP13/<br>AGAP00840<br>7	MKASKHPTTRGSDARTTPLLLLLCLFIASQANLLVQAQQSQPQSNDVNRYNPQDPFGGRQQYNPQYPSSQYDPNTRTNQGLPPSQSSGTNYQRDYYGNNAVNG<br>NNQDSNSIDSNRFGQDNRNVVLQSTTPRDYFNRQTTPPFRTRTSNRNPFSNNDYAAGNSFSQGITYFIVASKMVRPGQVYKVAVSVLESHLPLTVRTSISRDGVEL<br>SSETKPITVGVPETMLMRVPPTSVVGEYKLRVEGSYDKNFGGYVFANETKLIFSQRSMTIFVQTDKPVYMQGETVRFRAIPITTELKGFDKEMNVYMLDPAGHIM<br>RRWLSRQSNQGSVSLQYQLSDQPVFGEWKIRIEAQGQIEEARFNVEEYYQTRFEVNVTMPAFFFNTDRYIYGRIMANFTNGTPVKGNLTLKATIRPIGFFNPEAIN<br>QMNRVGNLGRLTNLNQNVPYYLQKTNPDLLNQYNPQNSFSSQIGGRDQGGYDGVGQGYDGRYSSNNFQDSYVIERHLNFDEEWPFWIKKPVESDSQWDSWTN<br>TYRDSLPYLRFFNGTYNFRFPMAELAQLVPNLSGMEILFTARVGERYYNEIVEGYSLTRVYNSSIRIAFLGDSPQVFKPSMPFTVYLIAEYHDGSPLPLDEFNAGRM<br>EVSGTIDSRASGGRNSFDSRVLHMSQKAGVWELKLDIRHDLNLENTKQTNEFLNEIQSMRLVANYIDPSGERASTELLLLSHFSPNNHNIKVHTSTADAKVGEYIT<br>LHVQSNFYIKDFNYLVMSKGIILVTGHENMKGGVKTMAITLSAEMAPAATIVVWHIGRYGKLLTDSLTFPVNGISRNNFTVFINNRKARTGEKVEVAIYGEPGSY<br>VGLSAIDNAFYTMQAGNELTYANVIQKMLSFDEHTNGTFKKTWMSHDGDPDELVYYPASTFGIDANRTFDYAGLVVFTDGVVPMRPTICDAALNYSECLNGRC<br>YRTDKRCDGYMDCEDGTDEAGCSTRNETLLVEFRKFRFNRILRHYQNVWLWKDVNIGPHGRYIFNLDVPQVPALWSVSAFGISGTRGYGMIRKPIEYVGIQPFFI<br>NVEMPTACHQGEQVGIRVAVFNYQTVDIEATVVLHSSPDYQFVHVEEDGIVRSYNPRTSFGEHQFYIYLNAQDSTNVYLPIVPTRLGEIEVTIHASTLLGAHQISRK<br>ITVEADGLPQYRHQSILLDLRNRAYLVQFMHVNVTETPIIPYEIDRYYVFGSNRARISVVGDVVGAIFPTMPINTTSLLELPMDAAEQNMFSFAANFYTIKYMRAST<br>LRDKRTEKQAFHFMNILYQRQLSYLMEDGGFSLFRADWNQSAPSVWLTAYCAQVFGEMAGLYEYENFIFIDPYMIQKNMHWLLRHQKEDGSFWEETWLPDRK<br>ANSSAFFSRNDVVREKNVTLTAHVLITLTTVNLPAGRLAARVALSQQRALQYLQRNLATIKESGSTYEIAIVAYALMIAKAPKAEAAFTMLSAKMRSIGEFNYWG<br>EEEVPLPPTKLENQRYFSLPRLPYKYDSLNIQTTAYALLTYVSRQEIHVDPIVAWLNAQRLTDGGWASSQDTGMAMKALTEYSTRNRVSNVTQLAIKVEATSLPG<br>ETKQLYITRKSLAQQQFLDVPNAWGTVRVEATGVGYAILQMHVQYSVDTYKFQTQPPVPAFDLTTRTIFHGRNQSHISYVICQRWINTQESIRSGMAVLDVAVPT<br>GYMIQQQKLDSYILSQRVRNLQRARFQERKVLFYFDYLDNDYVCVNFTLERWMPVANMSRYLPIRVYDYYAPERFNETIFDSLQTYLLNICEVCGSSQCPYCSIY<br>NIAVRFPGSIVLMLLTGIVLVARHYRIVNPNGWIFLND
AgTEP12/<br>AGAP00865<br>4	MCFRVAIYCLLLGTATKRVLADEGDYITIFTTRSWVENEDVSIILANMDNAPGTFEIENIGMEQGDVLVSTTVDPKHVQLYVIPSEWSATGEEAIQLDIVTSAGQPE<br>EARLTGQIERTAQPKVLIQLNDVFHTPGDFLKYRILLTDGLNKPLGRTKRPFNLTIVLEHDTQQTVVSRDVQLYPGEIYSGEHLFVDDRDLGNWTVAVTIGEQTTS<br>KQFQVILYSAPVHQITIDTGELITLQDTEIAITIDAMYSFGKPIRGTLQMKVYSDEDHVVERTVSIAGRKMVQIPMQEVVPKGSPPLETKTLHINATVTTTNGLSQRT<br>YNQARAIPMYASPYKLVVERTVGFTPGQNATVFLKILRANGASLNGLEDDAEMVSFTTDDSGEVFTANLEYRNMSASITLEPAYSPQLYVRILQPKQYRKRDAF<br>KLAIVASYAMDGVLTIIRKHNGENVPHYIECSMQNYQELFVPTVRLQDVKRVYVFARFEGTLMQASASYKEPALSEKVHLSVDEGSIKVFSNEDASRVGIAVYEG<br>SLDAAQLKNIYTRAMFNGTTYPKIEEIFPLSINDLIVSPLPEQGEVDKQQNPKSINRLLLWEEGTISNRQAVFQFVPPPHINQMIVTAFVFSPTGGLGIAEPIQWQRQQ<br>DIEIYLHIPYSAKKLEPVTVDVYIVNNRNEKVDFVLVELLNYANEFHFLNNSGRIDATKKTVYGRLRPNEVQRTEFLIRPKKLGSITLRANAYTESNLFARAETILR<br>TISESVRRTDSIVRLFNVDNSSQQLDDIKLPIPRTVDPGTEKITITLDRQQTEMASLTTSMLLDKLIQTDPLTMAMRAALTLEVLALGGLQWNERKALAERQMNESI<br>TKIMAYKNVDGSFTIPDHHTPSSACWDTIIAVQALTFANDRLASASLAETILKTLQWIKSKQAPDGHFCTDDGEQSELERVDKTAHVLLLLLHMKNYIMRHVSAI<br>DRARNYLLSSTSTLHSSYHLALAGHVLQLSLERPTGTEDRTMINRQASHILIGILQARQQSASGSKVWFNKPSSTPDLEATAYALMLMTSKKFLYDAGPIVNWMK<br>EQPYRRVVNASITPNSHIALRALIDYAKHTTFLEKRYIAKVTAKDRTGVIARHELTHRDIGSQVWILPSTTRTVSFTIEGTISGALRIHYSYMESVTLQGKKFNIDVL<br>RYGTSNEDYTDWRVCLRFLPKGFYEKTRMVTCEIAFPTGYIALDDSVDELNQLDGVVATVLRNDETQLSITFEEIGVQQKCFNVSGFRQNIETRQLPGTIKVFDVT<br>DESNVAFKQLDTKT
AgTEP4/<br>AGAP01081<br>2	MRHLASASLLVITLLINFVHGILIVGPKHIRAGQNYTVILSSFESNTNERNLLLQLDGLSAGGKKLLHLGQPTRVQPLSNSVISFPLPESLPAGSYKLTIDGQNGFKFH<br>READLLLAASTTTALVQLSKPIYKPGDVLQFRVIVLGGDLKPPAPSVTATVIVHDPQRNVIRRWTAVSLQLGVFEEQLQIGTVPLLGRYTITVTVTGANEIVSKTFD<br>VREYVLPAFEVAVKARAVPLEKHQRLNLTLSARYYTGQPVRGVATVELYLEDDKLDQRRVVGVYGAIQLDLPFNEHLSVYDDSQDVRVHVAFTQDETNRTIYK<br>EQRITVYKLPYRVELVKEQPEFRAKVPFDCQLRVRYQDGTPAKGAAFEVKVEGAYTTDRRVAYTSDAAGVIKLTLQPEASSESIDITVNAGSEELLYERISKREAS<br>KAFLQVKLDSDVKIGKPFKLKVTCNEELSFFMYYVVSRGVIVDSAFLQPKKVTEHFIEIEASDQMVPRSKVIVVTVAKNVVLCDFVDIDFEDLRNNFDLQIDRTEI<br>RPGDQLQLNMRGPPGAFVALAAYDKSLLQYSNNHDIFWEDVWGVFDKFYSVERNEFDLFHSLGLFARTMEHITFDKANDQTARDGSSSSKNGPSNSQASFRTNF<br>LESWLWKTDKIGSSGSATTKESVPDTITAWHLTGFSIDPVYGLGIIKQPLQLTTVQPFYIVPNMPYSIKRGELVELQFIVFNNFPKKYKASVTLFSVDNQTEFVGRP<br>ATETSYTKSIEASPDTGVPVAFLIKARKLGEMTVRVDASIEPAKDSIESVIRVIPESLVKREMISRFFCHNTYQNQSFVLGLDFDRKADAGTRKIDFILTPNILTSVMD<br>NLESLLSVPTGCGEQNMMRLVPIVLVLDYLTSIGSADKQLTAKAIGLLRAGYQNQMRYRQPDGSFGLWEKSGGAVFLTAFVGKTLATAAKYISEIEPSMVEQAF<br>DWLAARQHSTGRFDEVGPVFHRDMQGGLRQGIALTSFVLIALLEQPKVATKHRAAIEKGIDYVTQTLGSIEDSYDLAIATYALLLQKHSSGERFLEKLIGLSTVQQ<br>NGTERFWARDAHGIETTAYGLLSFVLAEKYVDGTSIMRWLVKQRYTPGSFPRTQDTFVGLKALTKLAEKISPSRNDYSVQLRHAGRKKEFRVTSQDIGTLQNAQ<br>QGVDETAQLELHVAGIGFGLLQVVYEYGVDLRNFTAQFVLELQKSVTNANHQLQLEVCSSFTPQLSDGRSNMVLVEVNFPSGYTVEQRGQPITGATKHNPIQKT<br>EVRFGATSVVVYYNSMGPERNCFTITAYRRQKVTLKRPAYVLVHDYYDPKLNAIKMYQVDD
AgTEP18/<br>AGAP01081<br>3	MRQFIRSRILTVIIFIAAAHGLLVMGPKCIRDNQNYTLTISNFYSNPSKLDLMVKLEGKNDNGLSVLNVTKMVDVPCKSIRIIDFSIPDSLSSGNYKITIDGLQGFNFH<br>EEAELVYLSK
AgTEP6/<br>AGAP01081<br>4	MSKSIMWKCIRSRILMVIIVIGAAHGLLVVGPKFIRANQEYTLVISNFNSQLSKVDLLLKLEGETDNGLSVLNVTKMVDVRRNMNRMINFNMPEDLTAGNYKITI<br>DGQRGFSFHKEAELVYLSKSISGLIQVDKPVFKPGDTVNFRVIVLDTELKPPARVKSVYVTIRDPQRNVIRKWSTAKLYAGVFESDLQIAPTPMLGVWNISVEVEG<br>EELVSKTFEVKEYVLSTFDVQVMPSVIPLEEHQAVNLTIEANYHFGKPVQGVAKVELYLDDDKLKLKKELTVYGKGQVELRFDNFAMDADQQDVPVKVSFVEQ<br>YTNRTVVKQSQITVYRYAYRVELIKESPQFRPGLPFKCALQFTHHDGTPAKGISGKVEVSDVRFETTTTSDNDGLIKLELQPSEGTEQLSIHFNAVDGFFFYEDVNK<br>VETVTDAYIKLELKSPIKRNKLMRFMVTCTERMTFFVYYVMSKGNIIDAGFMRPNKQTKYLLQLNATEKMIPKAKILVATLVNRTMVNDIVDIDFQGFRSNFDLS<br>IDEQEIKPGRQIELSMSGRPGAYVGLAAYDKALLLFNKNHDLFWEDIGQVFDGFHSYDTNEFDLFDNMGLFARMDNIMFDQSNDKSARSGQQADGTVFRKQFLE<br>SWLWKTAIIGNSGTLKLIEVVPDTTTTWYLTGFSIDPVYGLGIVKKPIEITTVQPFVVMESLPYSIKRGEAIEIQFILISNLQEEYTVDVTLYNENNEMEFLGRSISNVS<br>YTKSVSVPPKVGKPVSFLVKAKKLGEMMVRVKASIANELATDALEKVIRVTPESLVQSGVESFGFFMDTHQNRTFLVNPNIDKKADNGSIAIELRVNPNLLIKVK<br>ENLNDLRTVSPTCKNVIRLALNFAVADYLIACGPKEQNLPENAVDALSKEYMLLMTCLNSDSSFDSVQNIVSNIFYTAFVANTLDTAPKNVCQTSNVKLEKAFD<br>WLASRQQRFGSFREIETDLHYTRSEIALTSYVLASMLENESAKVKHAVVIEKGMWFLSNQFDLITSANDLAIVTYAMMLYGHWRKDAAFEKLIDMSTITNNGAE<br>RYWKTSNSVEATSYALLSHVLSNKLLAALPIMRWLVNQKSEIDSVSGQETTYLRLKALSRMTNKISPSRNDYMVQLKYKQSTRLLRFDSYRNMIQNITTPQDLRK<br>IKITVEGIGVGLLEVMYKYRLNLVNFEHRFQLDLQKQNTSSDNELRLKVCANYIPTLRDSHSNMTLIEVTLPSGYVVDRNPISEQTTVNPIQNMEIRYGGTSVVLY<br>YYNMGTERNCFTVTAYRRFKVALKRPAYVVVYDYYDTNQNAIKVYEMDKQNVCEICDACGCTAECKT
AgTEP1/<br>AGAP01081<br>5	MWQFIRSRILTVIIFIGAAHGLLVVGPKFIRANQEYTLVISNFNSQLSKVDLLLKLEGETDNGLSVLNVTKMVDVRRNMNRMINFNMPEDLTAGNYKITIDGQRGF<br>SFHKEAELVYLSKSISGLIQVDKPVFKPGDTVNFRVIVLDTELKPPARVKSVYVTIRDPQRNVIRKWSTAKLYAGVFESDLQIAPTPMLGVWNISVEVEGEELVSK<br>TFEVKEYVLSTFDVQVMPSVIPLEEHQAVNLTIEANYHFGKPVQGVAKVELYLDDDKLKLKKELTVYGKGQVELRFDNFAMDADQQDVPVKVSFVEQYTNRT<br>VVKQSQITVYRYAYRVELIKESPQFRPGLPFKCALQFTHHDGTPAKGISGKVEVSDVRFETTTTSDNDGLIKLELQPSEGTEQLSIHFNAVDGFFFYEDVNKVETVT<br>DAYIKLELKSPIKRNKLMRFMVTCTERMTFFVYYVMSKGNIIDAGFMRPNKQPKYLLQLNATEKMIPRAKILIATVAGRTVVYDFADLDFQELRNNFDLSIDEQEI<br>KPGRQIELSMSGRPGAYVGLAAYDKALLLFNKNHDLFWEDIGQVFDGFHAINENEFDIFHSLGLFARTLDDILFDSANEKTGRNALQSGKPIGKLVSYRTNFQES<br>WLWKNVSIGRSGSRKLIEVVPDTTTSWYLTGFSIDPVYGLGIIKKPIQFTTVQPFYIVENLPYSIKRGEAVVLQFTLFNNLGAEYIADVTLYNVANQTEFVGRPNTD<br>LSYTKSVSVPPKVGVPISFLIKARKLGEMAVRVKASIMLGHETDALEKVIRVMPESLVQPRMDTRFFCFDDHKNQTFPINLDINKKADSGSTKIEFRLNPNLLTTVI<br>KNLDHLLGVPTGCGEQNMVKFVPNILVLDYLHAIGSKEQHLIDKATNLLRQGYQNQMRYRQTDGSFGLWETTNGSVFLTAFVGTSMQTAVKYISDIDAAMVEK<br>ALDWLASKQHFSGRFDKAGAEYHKEMQGGLRNGVALTSYVLMALLENDIAKAKHAEVIQKGMTYLSNQFGSINNAYDLSIATYAMMLNGHTMKEEALNKLID<br>MSFIDADKNERFWNTTNPIETTAYALLSFVMAEKYTDGIPVMNWLVNQRYVTGSFPSTQDTFVGLKALTKMAEKISPSRNDYTVQLKYKKSAKYFKINSEQIDV

	ENFVDIPEDTKKLEINVGGIGFGLLEVVYQFNLNLVNFENRFQLDLEKQNTGSDYELRLKVCASYIPQLTDRRSNMALIEVTLPSGYVVDRNPISEQTKVNPIQKTE<br>IRYGGTSVVLYYDNMGSERNCFTLTAYRRFKVALKRPAYVVVYDYYNTNLNAIKVYEVDKQNLCEICDEEDCPAECKK
AgTEP3/<br>AGAP01081<br>6	MCHPLVVMPWYVRILVVISLLGSSWGVLVVGPKFVRSNQEYALVISNFNSGSSKVNLMLHMEGFSKNQTSVFAIRKPVDVRRFMSRIVSFDIPNIASPVDIKLTMV<br>GQRGFSFHEEEHLVHRSKSISGLIQIDKPVFRPGDLVKFRAIVLDTELKPPARIKSVNVTIQDPHQNKIRGWPAAKLYAGVFENDLQLAPAPLLGVWNITVQVGEE<br>QLVFKTFEVKEYVLTSYDVQVMPSVMPLVEHQTLNLTIVANYHFGKPVQGVAKVELYLVDDTLDQKKELTMYGMGQVELRFNELLELYEDQQDVRVKLTFTE<br>QHTNRTVVKEQAITVYKHPYRAQLTKESPQFRPGTPFKCTLTLIYHDGRPAGHVPFFVNVEGEDVDHQQTYTTGRDGTIKLLMRPTELTETIDITVSEDNSEFTYT<br>ERIEKVHADTNVFLKLELKSPIKLGKLIRLMVTCNERMTFFIYYVISKGNIVDAGFVRPNRQTKFMFQLTASEKMIPKAYIFVATVSQDVVVWDSLEIDLKQFSNH<br>LDIIIDEKELKPGQEIELLLKGRPSAYVGLAAYDKGLLAYSKQHDLFWEDVMQVFDTFHATDQNEFDVFNSMGLFARLSGGNRIGASPTTTERFGSAASRPISRLV<br>AYRTNFLESWLWQNVSIGRTGSRTVHEVLPDTTTSWYLTGFSIDPVYGLGIIKKPIEFITVKPFYIVDSLPYSIKRGEAAVLQFTLFNNLEAEYIADVTLYNVANQTE<br>FIERPDKDLSYTKSVSVPPKVGVPISFGVKARKLGEMVVRIKASIMTGKETDAMEKVIRVIPENIMFEKTETRFFSMDEYGKQEFNMQLDIPKNISTVQIKCRISSNL<br>LSPVIHNLDSLLDVPSASGAPSMINFIPPLVVLDYLKAVSSTTTHLIEKATGLLRNGYQLELKYRQRDGSFGNWRDSKGSVFVTALVGTSLEAASKHITEVDLTLV<br>DRLFEWLAAKQHSSGRFDEEQPITYYSLQGGSRNGIALTSFVLIAFLQNTKASAQHRSIIEKGIQYVANQLESIADVYDLSLATYALMLADHRQKSSALNKLIELGI<br>ATNETRYWPRDTASIETTAYALLSLVHAKRYADGLMVMHWLVNQQSATGSFPRTQDTFVGIRALAALSEAIAPQKNDYTAIVLHGKARKVYKVAASEADQEY<br>HDVLPGDSKLVRFSANGRGFGMFTVAFQYGIDVRNIEHGFSLRLVDQFSNEAYTLQLQVCTSFSPQLMHTRSNLALVEVNFPSGYVVSRKSLVDETRRNPFKDVE<br>VRYGQTSLVIYYETLGPEENCFSVTANRLFRVAFHRQAYVMVHDTYDEKFRAIKFYQVPHDGADIQSYLD
AgTEP11/<br>AGAP01081<br>8	MWQFIRSRLFSVIICTGAAHGVLVVGPKYIRTNQEYTLVISNFNSNQTKVDLIVKLEGETDNGLSVLNFTKTVVVRRNMNQMININMPESLAEGNYKITIDAQQGF<br>SFHQEAELVYLSKSISGLIQVDKPVFKPGDTVNFRVIVLDTELKPPAKVKSVHVTIRDPQRNVIHTWSSAKLYTGVFESNLQIAPTPMLVIWNILVQVEGEELVSKT<br>FEVKEYVLSTFDVQVMPSVIPLEEHQAVNLTIEAYYHFGKPVQGVAKVELYLDDELIDQKKELNVYGKGQVELLFFGKFEMYEDQQDVQVKVTFIEHYTNRTV<br>VKLSQITIYKHAYRVELIKESPHFHPGIPFKCVLQFTYHDGTPATGITGKVEVSGMGIETTATRDNDGLIKLELQPNQDIESMHVSISVSIFVDLMNYNYKSLLILQD<br>VFATNAYIKIELKSPIKLNKLMRFTVTCTERMTFFVYYVVSKGNIIDAGFMRPNKRTKYLLQLNATEKMIPKAKILVATLVNRTMVNDIVDIDFQGFRNNFDLSID<br>EQEIKPGRQIELSMSGRPGAYVGLAAYDKSLLLFNKNHDLFWEDFLELFDGFHSYYTNEFDLFHNMGLFARMDNIMFDESNDKSARSGQQADGTVFRKQFLES<br>WLWKTAIIGNSGTLKLIEVVPDTTTTWYLTGFSIDPVYGLGIIKKPIELTTVQPLIVMESLPYSIKRGEAIEIQFILISNLQEEYTVDVTLYNENNEMEFLGRSISNVSY<br>TKSVSVSPKVGKPVSFLVKAKKLGEMMVRVKASIAIGLATDALEKVIRVMPESLVQSGVESFGFFMDSHQNRTLYR
AgTEP10/<br>AGAP01081<br>9	MWQFISSRILTVIVFIGAANGLLFVGPKYIRDNQNYTLTISNFYSNPSKMDLMVTLEGQIDNGLSVLNVTRMIDVQRNSIRMISFSIPDNLSSGNYKITIDGRQGFNF<br>HMETELLYLKKSVAGLIQIDKPIFNPGDKVNFRVIVLDTELKPPARVKSVHVTIRDPQRNVIRKWSTAKLYAGVFEGDLQIAPTPMFGVWNILVQVEGEELVSKTF<br>EVKEYVLSTFAVQVMPSVIPLEEHQALNLTIEANDYFGKPVQGVAKVELYLDDDMIDQKKELTVYGTGQVELLFFDKFEMYVDQQDVRVKVTFIEHYTNRTVV<br>KQSQITVYKHAYRVQLIKGNPHFHPGLPFKCVLQFTHHDGTPATGITGKVEVSGIGFGTTVTSDNDGLVKLELQPSEGIESIDVSFLNNQNGLIFEETMYKEESYTN<br>AYIKLELKSSINLYKLMRFMVTCTERTTFFVYYVVSKGNIIDADFIRLNKEMTYHLQLNATEKMIPKAKILIATVVGRTVVYDCMDLDFQEFRNNFDLSVDEQEIK<br>PGRQIELSMSGRPGAYVGLAAYDKALLLFNQNHDLFWEDFLKVFDGFHSYRENDYDLFHSMGLFAKTLDDILFENSNHKSERSGQQMEQTVVRKQFVESWLW<br>KNVTIGSSGSLKLTEVVPDTMTSWYLTGFSIDPVVGLGIINNPIEFTTVQPFFILECLPYSIKRDEASEIQFIVVSNLQEGHTVDVTLYNENNEMEIIGRSIANVNYTKS<br>VHVLPKVGKPISFLVKAKKLGEMMVRVKASISNGIATDALEKVIRVMPESLVQSRVESFGFFMDTHQNQTFLVNPSIDKKANKGSVKFELRVNPNLLITVQENLD<br>NIRADISGCSESIKDMLHFVVHDYLVAIGSTHPYESNDNKCIKSNGSWRNQVFETAFLVNALQDAAKYIYFTDRLKREKAFDWLASQQHHSGSFKETETDLHYKR<br>SDVALTSYVLAVMLENESAKVQHAVVIEKGMSFLSDQLDLITSANDLAIVTYAMMLYGHRLKDAAFEKLIDVSTITNNGAERYWNTSNSVEATSYALLSHVVA<br>NKLLAALPIMRWLVNQRSDLDSVAGQENTYLRLKALSRMTNKISPSRNDYTVQLKYKQSTRLLRFDKYSNMIQNITTPQGVRKIEITVMGIGAGLLEVIYQYSLN<br>LTNFENRFQLDLQKQKTSSDHELRLKVCVSFIPTVSESRSNMALIEVTLPSGYVVDHNPISEQTTVNPIDHFEIRYGGASVVVVYYKNMSNVRNCFTVTAYRRFKVA<br>LKRPAYVVVVYDYYDTNKNAIKMYEVDKQNVCEICEEDDCPAECQK
AgTEP9/<br>AGAP01083<br>0	MWQLIRSRILTVIICIGAAHGLLFVGPKFIRDNHSYTLTISNFYSNPRKMYLMVKLEGQTDNGLSVLNITKMIDVRSNSIRMISFCMPDNLSTGDYKITIDGQQGFNF<br>HMETDLFYLKKTVAGLIQVDKPVFKPGDTVNFRVIVLDTELKPPARVKSVHVTIRDPQRSVIRKWSTAKLYTGVFEGDLQIAPTPMLGVWNILVQVEGEELVSKT<br>FEVKEYVLSMFDVQVMPSVIPLKKHQALNLTIEAYYHFGKPVQGVAKVELYLDDDKLDQQKEITVYGIGQVELRFADYFDMYEDQQDVRVKVTFIEHYTNRTV<br>VKQSQITVYRYAYRVQLIKESPQFRPGLPFKCALQFTHHDGTPAKGITGKVEVTDVEFETTATSDNDGLIKLELHPSEGTEYLGVNFDSIDGFYYYEGVSQIQTDA<br>FIKLELKSPINLNKLMRFIVTCTERMTFFVYYVVSKSNIVDAGFVRPKNETTFLLQLYATEKLFPKAKMLVATVTGRTVVYDYINLDFQVFHNNFTLSVDEQEIKP<br>GRQIELSMSGRPGAYVGLAAYDKALLLFNQNHDLILDDFLKVFDGFHVHHEGEFDQLHTMGLFARTLDDFLFQNYNHKSERNGQQMEQTVVRKQFVESWLWK<br>NATIGSSGSLKLTEVVPDTTTSWYLTAFSIDPVVGLGIIKKPIEFTTVQPFVIMESLPYSIKRGEAIEIQFILISSLQEEHTADVTLYNENNEMEFIGRSIANASYTKSVR<br>VLPKVGKPISFLVKAKKLGEMMVRVKASIANGIAADALEKVIQVTPESLVQSGVESFGFFMNTYQNRTFLVNPNIDKKADNGSVEIKLRFNPNLLITVKDNLNDIR<br>TDWSRCNEGIRGTLNFVVHDYLNTIGSSDQISSDDSAKIIIHFVRLQKCFISKAPWRNKVFDTAFLVNALHNAMKYVYWNDKHKLEKIFAWLASQQHHSGSFKET<br>ESDLHYKRSEVALTSYVLAVMLENESAKVEHAVVIEKGMSFLSNQLDLITSANDLAIVTYAMMLYGHRLRDAAFEKLIDMSTITNNGTERYWNTSNSVEATSFA<br>LLSYVVPNKLLEALPIMRWLVNQKSELNSVSGQQNTYLRLKALSSIAKKISPSRNDLVAKLKYKQSTRLLRFDKYSNMIQNITTPQGVRKIEITVMGIGAGLLEVIY<br>QYSLNLMNFEHRFQLDVQKQNTSSNHELRLKVCASFIPTVSESRSNMALIEVTLPSGYEVDHNPISEQTTVNPIYHIEIRYGGTSVVVYYKNMSNIRNCFTVSAYRR<br>LKVALKRPAYVVVVYDYYDTNLNAIKVYEVDKQNVCEICEEENCPAECKI
AgTEP8/<br>AGAP01083<br>1	MMERWLKAFPHLEGSVLLQFDAVDAFLFYEDVNKVETDAFIKLELKSPIKLNKLMHFTVTCTERMSFFVYYVVSKSNIIDAGFMLANKETTFLLQLNATENMIPK<br>AKILIITVAGRTVMYDYINLDFQELRNNFDLSVDEQEIKPGRQIELSMSGRPGAYVGLAAYDKALLLFNKNHDLFWEDFLKVFDGFHSYDTNEFDLFHTMGLFAR<br>TLDDFFVQNYNHKSERNGQQMEQTVVRKQFVESWLWKNVTIGSSGSLKLTEVVPDTTTSWYLTGFSIDPVVGLGIIKKPIEFTTVQPFFILESLPYSIKRGEAIEIQF<br>ILISNLQGEHTVDVTLYNENNEMEFIGRSIANASYTKSVSVLPKWGKPISFLVKAKKLGEMMVRVKASIANGLATDALEKVIRVTPESLEQSRVESFGIFFDEYQN<br>QTFSVFPNIPKNADNGSVEIELRVNPNLLITVKENLNDIRTDWSECSKGIRFTLNFAVHNYLVAIGSSDQISSDDNATLSKHYIRLEKCFNSNGSWRNKVFDTAFLV<br>NALHNAMKYVSWKDKRKLEKAFDWLASQQHHSGSFKETESDLHYKRSDVALTSYVLAVMLENESAKVEHAVVIEKGMSFLSDQLESITSANDLAIVTYAMMS<br>YGHRLRDAAFEKLIYMSTITNNGAERYWNPSNSVEATSFALLSHVVANKLQGALPIMRWLVNHRSELNSVSGQQNTYLRFKALSSIAKKIALSRNDYDVRIKYRL<br>SRKLFRFTSYDSDTQHMLITQNVRKLEIHVGGIGAGLLQVFYRYNLNLMNFEHRFKLDLQKQITNFDQELKLNVCANYIPTVSESRSNMALVEVTLPSGYVVDHN<br>PISEQTTVNPIQRIEIRYGGTSVVLYYYSMGSERNCFTVSAYRRFKVALKRPAYVVVVYDYYDTNLNAIKVYEVDKQNVCEICEEEDCRAECKI
AgTEP19/<br>AGAP01083<br>2	MMERWLKAFPHLEGSSLLQFDAVDAFLFYEDVNKVETDAFIKLELKSPIKLNKLMRFIVTCTERMTFFVYYVVSKSNIVDAGFVRPKNETTFLLQLYATEKMFPK<br>AKMLVATVTGRTVVYDYMYLDFQVFHNNFTLSVDEQEIKPRRQIELSMSGRPGAYVGLAAYDKALLLFNKNHDLFWEDFLKVFDGFHSYDTNEFDLFHTMGLF<br>ARTLDDFFVQNYNHKSERSGQHMEQTVVRKQFVESWLWKNVTIGSSGSLKLTEVVPDTTTSWYLTGFSIDPVVGLGIIKKPIEFTTVQPFFIVERLPYSIKRGEAIEI<br>QFILISNLQEEHTVDVTLYNENNEMEFIGRSIANASYTKSVSVLPKVGKPISFLVKAKKLGEMMVRVKASIANGLATDALEKVIRVTPESLEQSRVESFGIFFDEYQ<br>NQTFSVFPNIPKNADNGSVEIELRVNPNLLISVKENLNDIRTDWSECSKGIRFTLNFAVHNYLVAIGSSDQISSDDNATLSKHYMRLEKCFISNGSWRNKVFDTAFL<br>VNALHNVSIYVSWKDKRKLEKAFDWLASQQHHSGSFKETESDLHYKRSDVALTSYVLAVMLENESAKVEHAVVIEKGMSFLSDQLDLITSANDLAIVTYAMML<br>YGHRLKDAAFEKLIDMSTITNNGTERYWNTSNSVEATSFALLSHVVANKLLEALPIMRWLVNHRSELNSVSGEQNTYLRFKALSSIAKKIALSRNDYDVRINYRL<br>RRELFRFTSYDSDTQHMLITQNVKKLEIHVGGIGAGLLQVFYRYNLNLMNFEHRFKLDLQKQITNFDQELKLNVCANYIPTVSESRSNMALVEVTLPSGYVVDHN<br>PISEQTTVNPIQNIEIRYGGTSVVLYYNSMGSERNCFTVSAYRRFKVALKRPAYVVVVYDYYDTNLNAIKVYEVDKQNVCEICEEEDCRAECKI
CqTEP26/<br>CPIJ000345	MLNYLARRHLYGTRTGSPFPVWTSLLRADDQFCVERRRRNSRIRLVVLNPRLESGFLKMPRLRLDRLWFLIVLVWGSMIGLCQSQSLSILGQKTIRPFAPYTVSIT<br>NTLSKDVQLEILLYGSDLDDKQTIKAKRESEQRITFETKHVTDGEYHLSIRSLDHSVSFREDIELISDPRVFSIFIQIDKPVYKPGDMMHFRVIVIDSDTRPVTNLSYV<br>TVGLKDSTDTSIREWQYAKLHNGVFESAVLLPSSPNLGKWLLTVESEQVQKIKEFEVKPYVLPKFWIKVYPTEVLLAGKNTIKLTVESEFTFGRPVAGVVKVDLY<br>FSKIRRNSEHSISKYFERMTMFEFFLNEEVDLDDDKEFADVFVNVTVTEKHTNTTLFIFEPIRVFRDEFDIALIKPWPMFRPGMPFPLQVSFKDRMGRTAGSGQTVT<br>TRVTYRLNNEEDRETDIVGTISSRGLLSLPNIVPPVTATEMNVQFKIFLDEKKYNPGQEAYIDVEATSDSYVAFHAIDQSVLLVNDSWNDLTKANVVKEFDSFGDN<br>EFDPFHSMGLFMQTTSKQDSGTAKLSRYGLGFQNAKEILHIRTIFPETWLWQNVSMEGKTDVSFSAQVPDTMTSWLVTGFALSPTTGLGLIHEPITFKVEKTFYIV<br>AHLPYSIKRGEVTVIQVMIYNFIGNTLITDVRLYNKNDEIEFVDQSSSDTMRRTKAVIMPGNSGRQVSFLVKAKKIGEIAVKIEAVNSLKQDSVEHILRVEPESYLSS<br>KNEQRYIEHQEYKSTQENITIDIPRYTLPGSIEIAFDLDPNFLSIPIKNINQLMQTPKEYGEANMLDFVPQVVALDYLTESKMDNENLKNKMINYIKTGYHNQLKYK<br>LNNGSFCNWESEEHKKGSIFITSLVAKSFKIAAKHVDGLISNEIVESALKWLASNQQSDGSFVEVGDKIHNGLQSKYALTAYVLAAFLEVGELANKYSANINSAIE<br>YLVKIHTKLVDPYDLALTAYALSLKQHEAGKRCLDNIIERSNQTGAVRLWNEGPFSTEIGSYVVLTFLQYYPVIEATPIVNWLTNKRSETGGFSSIQSTFIGLRAMA<br>QYALKASVDAYDYTITLRWKKLLAKKFNVNRYNARYTQTTIVPSDVRQVSVEIDGIGIGYYQVAYQYRTELKDAKHGFDLSLKLMEDSNYRVQNLKVCTKFRK<br>PESYTTSNLAYVEVFFPSGLHVNNENAITDLASGHKIMSKLSVAMTSHCIVLPMLLCMMPWITMCLL
CqTEP28/<br>CPIJ000346	MVTNRLKWLLQLVLCCGLFVELCQSQGSVEFVLIVPLRAAVMLWKEGIPQIARNPRSNSQSAQEEVIVRLSVIGPKLIRPFTPYTVAFANSLSRDARLEVVLEGPT<br>DSVSVLNRAARLAVGEIPNGEYRLSIKSLSADFGFNEEIDLIYDGKTESLFVQLDKPVYKPGDMLRFRVVVVDVNTRPVTNLKSVQVKLADKDGNSIMEWPFGRL<br>YNGVFETSFQLASSPVLGNWTLTALAGSSKVKKQFELREYILPKYYIKVYPTEVLLASKKQIKLAVETAYTFGRPLDGTLTVDLFLEDIYQRNPDHSVTKRIEGRT<br>SVEFDLKKEVEVEEDSIFANVWARVSVMETFTNKTETIMRSIPVYKNPYTITVIKSEPMFRARMSYNMQLEIKDHNEVPAKGGTTASITIMYDGGNFDEISMVGEV<br>DAKGMVPLELTPPTGATTLHMRVIYDSFEYDQFEEIYAAQSRSNQFITVTLNLKKYKVRPNKEVLFEVSCTERISHFSYILVARGSIVDSGNVNVFNKQKTSFRYR<br>LLPQLAPKAKLIVSYTNKEFLIFDELDLDFDVFNNDFEFSLDHEVGNDYHPGQDIYVDLKAANDSYIAFHAIDQSVLHLGRDGHVFSRDDVLEDLSQYGATEANE<br>FDPFHTMGLFLRTTAQVDFPYARQQLSRFGGSVFGKRVEKAIHIRTVFPETWLWRNYTMDGRNSKMTIASVVPDTVTSWTVTGFALSPTYGLGIMQETREFTVN<br>QPFYIIANLPYSIKRDEVAVIHVTVFNFLGNTLTTDVTLFNKNDEIEFVEKSSDDPTRRTKAIIVPGNNGKPISFLIKAKKLGEIAIKIQAVNPLKTDSVEHMLRVIPES<br>HMHEVNEARFIDLPKNTVQNFQIQVNIPREIDEGSARIKFTLDPDILGTAIKNLESLIRLPTGCGEQNMMRFVPNIVVLDYLSETGTIRDDIKEKAIGYLKSGYQNQM<br>KYKLSDGSFGIWASSRGGTFLTAFVAKSLKIADKYMSVDIDVVNKAFAWLAGKQQPDGRFTEVGDVAHADMQGGLRSTSYALTAYVLAAFLETKSIAQTHSDV<br>IRRSANYLMSNFDNMNNVYDLALTTYALSLQSPASSQKFFDKLVESSTYDSTNKVRSWSYKSLGVEIAGYALLSYVQREQILDATPIMRWLTSQRYDLGGFPGT<br>QDTYVGLKALAKFAAKASAHRNDYRVTVRPKKEKLLTFDVDKKVLSIQELDLDSSSRSIDVEVSGIGKGVFQVAYQYYQNILKEKSSFDLSVNLMNTTTYYRQQ<br>LHVCVKFNPKEAYQYSNMVLVEVFFPSGIVADDDAVQDLSSNRAIQKTELRFGGTSLVVYYTRLGVQKNCFTVTAERRFNVALHRPTYVVVVYDYYDNKQSDRF<br>AIQSYEGKVMQLCDVCEDEDCVTLACPPSTASG
CqTEP29/<br>CPIJ000347	MWVLELLILGICFIGYCKGQGFSAIGPNTIRPNTPYSISFTNSFPCHAPVDVTLQGGPVGCKLSINQTISAIVSRRTGKSVSFEVGNIAKGNYKVLVRSKDVGIPFNEE<br>IDLAYDGKTESIFILLDKPVYKPGDVLKFRIVVVDVNTRPASDIKTVNVTLSDANGNSIRRWPFGKLQHGVFESQVQLASSPTLGLWSFTVMTGRSKASKSLELRE<br>YVLPKYHVKVYPQRALLVTEKRIVLVVETAYTSGKPLEGSLTVDLFVDDASKRRPDYFTTKRIEGQTTVEFRLNDELEVESDSDVTFVFARVNVTETFTNRTESIV<br>ERIPVHRYPNTISVVKSDHLFSPGSLFSLQLIVQDHNGIPASDGQSATIKIIYDGDESGEESYEISLVKETDSKGVITVDIVPPRTAISFQMEITYDSIVYDNFEEIYAAQ<br>SESNQFITVTLNENRYKVRPNREVLFEISCTERMSHFAYTFWARGSIIDSGHVNVANKKRYSFRYRLLPQMAPKAKLIVTYTSKNFLIFDDLELNFDMFSNHFEFSL<br>DDDDYLPGQDIYVDLQAANDSYVAFHAIDQSVLHLGRNGHAFTQDDVLRDLKQYGVGESDEFEPFNVLLTAYARNHHSRSSRSSEKIDKAPTQIRTVFPETWLW<br>TNYSMDGKRSFMTIESVVPDTVTSWMVDGFALSPTLGLGVMKQPRVFTVNQPFYIIADLPYSIKRDEVVVIQVTVFNFLGTTLTSDVTLFNKNDEIEFVDNYVEFE<br>SATDVTLSDMNVRAVETQVTRRTKPVIVPANNGKSVSFMIKAKKLGDIAIKFEAVNTLKSDSVEHMLRVVPESHLHELNEARYIDLSETNYQKFDISINVPRNVDE

	GSVSVKFILDPDIFGSVVENLESLISLPCGCGEQNMIQLVPNVVVLDYMYETRTIKEELYSDLISKLTKGYENQLNYKLSDNSFAVFPEMRGCVFLTAFVAKSFAIA<br>SKYIYIDERVIEGALDWLVQTQQPDGRFEEIGDIIHVDMQGGLETSCFSLTAYVLIALLESKSVNGERWEILQKSADYLTTGLDSINNPYDLALATYALSLKDRNES<br>LPFLDKLVEFSQYDEATGTRSWSYHSLGVEIAGYALLSYIEHGLVPDATPIMRWLTTQRYDRGGFMSTQDTFVALKAMAKFSARVSTHRNDYRVAVIPKRDKM<br>YLFDVDSQSLNTLKQNLESTTRKVQVELLGKGAGIFQISYQYYQNIVVEKSSFNLEVNLLPNSTYYRQELNVCVSFNAKEAYEYSNMALVEVFFPSGLVADKNSV<br>RDLSIGRNIKKTELRFGGTSLVVYYIRLNAQPNCFVVSAERHFKVALHRPAHVVVYDYYDNGAQSDRFAIATYEGKVMQMCDVCDDEDCETLSCP
CqTEP30/<br>CPIJ000348	MAVSWSVRRIGQILLGCAAWIGLGHGQGLSVIGPNVIRPYAPFTVSFANSLSRNAQFDVVLMNSNDDSILNETNVMVPRRSGKSASLQVGNIPEGEYKVLIRGIDA<br>DGDFQEEVDLHYHSKTESVFIQLDKPVYKPGDVLRFRVVVVDENTRPATSIKSVSVTLYDPRGNMINRWPFGMLYKGVFESSVQLASAPELGTWNLVVAIGSNQ<br>ISKEIELKEYTLPKFSVKVHPAELLLVKDKRIKLNVATAFMSGNQVEGNLVVDLFLNDINKRRPDHTYSGRIKAQSTVEFQLKEELEVDGDSDVTHVFARVSVLET<br>LSNKTETIVEAIQVYRNKYTISVVPSEPMFEPGKLFTLQFSIKDHYGTPARGGKLATIEILFDGDYFEDGEDELTFEREPDDMGVITLKIVPPNSAASFQLQVIIFLYN<br>QDPATKTNRFIISRVRPNKEVVFEVSCTERFKHFSYTFVARGRIIDAGHVNVMNKKKYSFRYKLLAEMAPQAKLIISHISRDYLIYDVLALDFDVFNNDFEFFLDSD<br>NYNPGQDVYVDMRAVKDSYVAFHGIDQSVLHLGHGGHLFTRADVFSELDQYSAIHVDELDPFSTLGLFLRTTSEVDSPYAEVLHSRSRRSVDSRRPQHAIQIRTV<br>FPETWFWRNYTLDGRSTSMTIESVVPDTVTSWLVTGFALSPTLGLGIINQPRMFTVDQPFYIVANLPYSIKRDEVAVIRVTVFNFLGSPLTTSVTLFNKNNEFEFVE<br>KSSGDDTRRTKAVFVPANNGHAVSFLIKAKKLGEIAIKIEAVNALKADSVEHILRVIPESHLIRRNEARFVDLTKQRSASYDIAIDIPRNVDEGSVFIKFTLDPDLLG<br>CVVKNLDSLIQLPCGCGEQNMMKFVPNVVILDYLSETKTISKEIESKAIENLKKGYQNQLRYRNSDGSFSVFRGRSGGTFLTAFVAQSFKLASKYISIDTNVIDQAY<br>RWLLSKQQPDGRFVEVGSIWSAAIQGGLRSSCFALTAFVLAAILEAGNVRLQNEAKIQKTINYLTFNLQNIDNSYDLALTAYALSLVPDRRISKPFLDKLIEKSTYD<br>ETTGTRHWNTASYGVETAGYAVLSYIAHDMIVDATPIVRWLTTHRYGEGGYRSTQDTFVGLKALAQFAAKASYHNNDYRVTVRPKAEKVLTFDVDSHKLAVQ<br>ELELDSATRNVNVQVTGVGTGIFQISYQYNQNIIHRQSSFNLEVNVLPNSTYYRQELSVCVSFIAREAYQYSNMALVEVFFPSGIVADESSVRDLSIGRNIQKTELR<br>FGGTSLVVYYLRLNAQPNCFGVTAERHFKVALHRPAHVVVYDYYDEGEHSDRFAIASYEGKVMQVCDVCEDEDCETLSCQ
CqTEP3/<br>CPIJ001747	MFPKFSHRFYSIVASNLLRPNSEYHVSVNNLNIADTVRFRITLNNTDTGVPVASEDVNLGPGESRLIPFSIGDIPQSEYGLTAEGLSGFTFRNETRITYQAKSFSVLVQ<br>TDKAIYKPGDTVRFRVLVLDPNTKPLQKVDTIKVHITDGKSNRIKQWSDAKLVKGVFESELALSSAPVLGRWMVNVEVLEKTTTKEFEVDEYVLPKFEVTIESPG<br>ITTFKDGKVKAIVRAKYTYGKPVKGEATVSAYPDFRFHYVQPFERDVITRKTVPVDGKGSVEFELRDEIKLEGDYTRDIVIEAVVEEELTGRKQNATTKVKIYDK<br>RYKMELIKSADKFKPGLPYTAWLKASFQDGAPIQDSVNQVEVTQESGWPERNTTKRSYTLDQNGMAKLVVNTDIEADYVEFKAEYLGSVFYLGSISKGWVKTN<br>AYVRAKVLTEVPTINKDVTVDVSATVPMKYFSYQVLGRGDVIVGGTIPVPDRTLHTFRFPASFAMVPRAKLVVSFVQDDGELVSDNVEIEFGNDLQNFVITLSKA<br>ESKPGEDVDIVVNTNPDSYVGLLGVDQSVLLLKSGNDITSGQVFDELKMYEQPSYGYYRRKRFAPWRHYNTYDDFNDVGATIMTNANEPPHKFAYPVAFFGTG<br>LTPIPAPYFPSTNRPIISTPANSQASNVGSDIIVTVRKQFPESWIWENDSKSLSTYTILPANDTSSRLPPITYKVHRAPDGTPLYTVIEKQPRSQPEHVFKTNTRPPLAG<br>PYAFSRIPKPHKNIPRLFLSQEIANTWLFESAYSGFSGQKTITKKVPDTITSWIITGFSVNPVYGLGLTQQPRKLNVFLPFFVSTNLPYSVKRGEIVSIPVVIFNYMESG<br>QTAEVTFDNSEYEFEFADVENEIHENSKAETSRKKTVEVPSNSGRTVSFMIRPTKLGHITIKVTATTALAGDGVERQLLVEPEGLPQFVNKAAFVDLRSAPEVMKN<br>FTVEVPKNAVPDSTRVEVSVIGDVLGSTVQNLDSLIRMPYGCGEQNMLNFVPNIVVLDYLKGTDQLTSKIEQKAKKFMESGYQRELTYRHDDGSFSAFGNSDPK<br>GSTWLTAFVARSFKQAASHISVEEAIIDKALEWLSDQQASNGSFPEVGKVSHKDMQGGSGEGIALTAYTLIAFLENRNLLPKYQNIVNKAVDYVARNIDGLNDV<br>YALAIAAYALQLADHSSKDFTLSQLDGKATTDGDTKWWHKPIPESDSKNPWYGKPNSVNVEMSSYAMLSFLEAGLDTDALPIMKWLISQRNDKGGFQSTQDTV<br>VGLQALAKLAAKISSKNNDVTIVVSYNENQQREIKVNSENNLILQKFELPSTAKNVDIKATGRGFAIVSLGYKYNMNVTGEWPRFVLDPQVNKNSNQDYLHLSV<br>CTSFVPTTGQNSSNMAVMEVGFPSGFTADSDTLPSLENMDYIKAMELKDGDTIVVLYFDSLDRNELCPTISAFRTHKVAKQKPAPVVIYDYYDNSRIARQFYDGP<br>KSTVCDICENEDCGESCSIRSQKQRSPKDDNGTADVKAVSGAVTVGALSTMHVLVAVLLLKMFY
CqTEP23A/<br>CPIJ004127	MSIVVKYAIFAVLVSSAGFVRSYYVALIPKTIREHSSSLVVIANLGFDHDEQFGISQNGKAKLVDVPVNVTMTVKPKLLNGRNAVQVVDTRSGVTTDLDVTMDR<br>SSLSVYIQTDKPIYKPGDTIRFRVLVLDHELKPSAMLKSINVRLADSQENLIREWSFGELVQGVFQSELELANFVRLGTWTLTATVMNGKAKSKEITVDEYVLPKH<br>EIRMSSSKIAVMTDETLTLVVDAVYTFGKPIRGDLTVIAEDSGQQVQNSINGRAIVNFDVASLVEGKHFGKMFYITFTATIKELGTDQSYKKSVHIPIYRDSYRITFR<br>KSSENLTPGVYFRCWFTVTDPTGNPFQKPENLIVKATQGKTTITAKKPDQNGVVYLKFDDIEDYSQSLKLDVSYAGKTHFFSVEPLHEDDSFIQVSILTEDPEQGQ<br>PVKVLVTSSFDLIPFLYFVIARGEILTRARIHAKAGQKSTFSFPTTLGMVPSASVVVFAVHEGRLMQDVAHFRVTRLKNFVNVTLSEDETEPRRQLQIRVDSLPGS<br>MVGLLAVDKSVLLLETGNDITRQSLQEDGIDMESKDIEDLGFTVLTNAKPSVSLSGEVDIRICDDNYGDHVRKHFPETWMWTEPMAVSSSGSLSLTATVPDTITG<br>WSISAFAVNAQHGLGLIEQPVYLTVVKPFFVTVNVAYSILKTETALIEVFVYNYGIEQEHAEVTLQVDSEEFLLLNEEQQVIGGVRQSERVTIPVDGIERVQFFAKP<br>RRSDELTITVTASTASNKFDDVQRTVKVTSGGIQFYQNEARFIDVEDAAQRFDIGLAIPRTATNGSELIVFSLEGLVMGAALTNLDGLIRLPTGCGEQRLVKLVPTV<br>IALEYMSVTGTLGNSMKYRAVSFLRQGYQNQLEYKRKDGSFHVFMWDDHGSLMVTAIVAKTLRIAGKYITVDERIARNAYDWIQRQQRSDGSFSEAGRVISRR<br>MQGSLGDDVPLTAFVLIAILEHKNLAQKYRTVVEKGTGYLSKKLDGLTKPYHLAMVAYALQLAKHNRKQFALEKLIEVSKYKDNMRWWQDGEASIETTAYAL<br>LTYLNNGMYIDAGPLMKWLVSKRYDLGGFDNTQNTLLGLQALAEYSKLLSPSRNNYEVTISYGDGNRNSLKIQGMTTPISQNLTLPANTRKVSVLIVGTGTGVF<br>QVAYQYNQIATDDEPRFELSKTIRRSSSGSVLDLHVCAKFKPRKRFEVTNMVLMEVLLPSGYVVADKTIDKLRNNKKVMKIEKDYDDTRLVIYFDALSDVDATC<br>VEVFSLRKAIVLGQIAGMIKVYDYYDPDREAYEYFDANA
CqTEP27/<br>CPIJ006840	METFKEFEVKPYILPKFFVKARPAEEYVITARQVKLIVETSFTFGKPVNGNVTYDSYKQDEFGSIEASRSKSNHQLKVTYDKNHYKIIDSGYIGSHTSNFKYRLTKR<br>MAPKAKLVVMYLKDYLIYDALDLVFDEFDNKFDFTLDPSPKNAYMPGQEISVEFEAASDSYVALHAIDQSILQLSQTDHLFTRDDLIRELSRYDATDENAFDPFH<br>TMGLFLKTTLKIDSPYGEDKLLRFGLMSEKRFEQPIHIRTEFPETWLWSNYSMQGNRKREITAYMPDTLTSWTITGFALSPSTGLSIIKQPLVAKVSHDFLIVANLP<br>YSIKRDEVVVIQATVFNNLGTGLSVDVKLYSKSDEIKFYNDTLTSSHFKKKTVFVENNSGQSVSFFIKANKLGEILVRLEAVAMFHSDSVEHVLRVIPESLVHREN<br>EVRFIDLSHETVNYTIPINIPRKTDNGSVGIEFALDPSFLGTTYENLDKLLLLPTGCCEQSMVKFIPDIVILDYLDAIRATNEGTKTKAINFLNQGYQNQLKCKLNNG<br>SFAIFPRASDNSAEGSIFLTAFIAKSLKIASKHITVDGNIVADAFRWLASQQRSDGKFIDEKNIYMGEMQGGIRKTSFALTAYVLAAFLETEDIGQQYPSVVNKSTE<br>YLKSNFDNMDHPYDLAVTSYAMSMSKDSKGPEFLKKLIDNSTFDRSNTYRYWNHETLGVEIASYALLAKLNDRSQLIDSTPIMRWLNSQRSNKGGFVGTQETFV<br>ALKALAKFAVEANPNRNEYGVQVRGGDPDKILKTFRVQRDKINVIKFDVESSERSVFVEVSGIGTGYFQVFYKYHRSILHEKESFNLAVNVLKSTTYDHLHLKVC<br>VAFKPKDSTSVSNMALVEIALPSGIMATENPVEDLSERKDIKQTELRYEGTSIVVYYMNLSVMEKCFNVNAERRYKVAMHRPSYVVAYDYYHKKRVAIKTYEG<br>NVKQPCELCEGEDCDIFSC
CqTEP13/<br>CPIJ016572	WTNTDESIRSGMAVLDVAVPTGYMIQQQKLDSYILSKRVRNLQRARYQERKVLFYFDYLDSDFVCVNFTLERWMPVANMSRYLPIRVYDYYAPERFNETLFDS<br>LQTYLLNICEVCGSSQCPYCSIYNAAIRNPVSVVLLVAASVLIVARHYRIVNPNSWIFWND
CqTEP23B/<br>CPIJ020143	MVASDHHAPGEKFQLHVETMPNSTVGILAIDQSALLLESGNQITMKAIFDELLGRTGGEEEEEVALRARFGNYDGVEEDEYIKPRKVFAQSWLWNKLVNSGPEG<br>NLTITEPIPDTITNWQISAFSLNPVHGLGVLETPLTLHVTKPFFVMLNLPYSIKKSETATVEVSVFNYLDELMYVGVTLKNHRQEFEFVGNSEGRTDASYQTKNLV<br>LTPQSAKSVKFLVKAKKMGDIMIKVVAESETASDSVERFLRVMPESLPYNKKESRYIQLKDSDEESRMSIDLEMPKYLDAGSENISLTVYSDLLGSAADDLQNLIR<br>MPSGSGEQNVLKMVPNVVLLDYITETKAFDSRVKARTVRFLELGYQNQLKFKREDGSFNVMGKEDPQGSVFITALVVKTLKQASKFITIDDSVLEQAYDWLKN<br>KQNLDGSFNEGGDLTNRELQKNHSDEDTLTAFVMIAFLEDGSITNKYKSVVKKGTDFLAAKDLHLQSTYTLSLIAYTLQLANHPSKKSAFDELLLNSKSSKSPQA<br>RWWDAGSNSLEATSYFLLTTLLLGNYVDSQPIVYWLTKNQYYYSGTLENTQTTFVSLQALAEYAKRVSTLRNNYYVTLTCYDRRKLKLAQNYAIQINPQNSRA<br>PLTLTLPSETRSLDLTLSGTGNGMFTINYHYLSNILRLNPRFTVKVKTLNTTTDEYMDLRVCAKFRPREAYEKAALTLMEITFPSGYIAFDETVQELESSDVVKKV<br>ATKHDETTLVLYFDTIPEHRFQCVDVTGFQQSEVLQQAPGTVRVYDFYDTTLIAIQYFDGKSFIALVPTTIRDQSTVRVGLANVGSGSFERFDVTLERSVKSDNRK<br>DLKSSVSVAPNEVKSAPFKIQTDKPIYKPGDTIKFRILVLNQELKPVTKLKSVNVKLTDSKRNLIREWTYGKLDNGVFQSQLELANFVPLGMWNLKVTAKDGEV<br>KQKSLNVSECNVHLWEAYQGKANVTTKHLVINTNIDGRSKTGVELDKVLRGRPTSDVTWLDVDALLQEAGSDHQFRSSERIPIYKSHHKITLRKSSDYLLSDVPY<br>KCWLILTDPFGKPLENPGVLKIKATRVIRFWVDKKYEMKKTPDRYGVVDLEFETSSSTEGLEVEVSYNGEITKFLVEAHSDDQRSFFQTTSLTERPKLGQPAQILV<br>VSSFKMSFLAYYVIARGEIASSGVVEVKASRSATFTVTPNAVMTSSSVAIFTINNGDLMQSIVHLKVNDLNNFVNATLSKNVTEPREKLTLNVQSKPGSMIGLLAV<br>DESVLLMEKGNDITKRSLSEDLQPSKDSDELEDLGFTVVTDACVKVNLISSRFGVLDVEIGPDFHRKEFPESWLWTDMATVNTSGQLEFSAIVPDTITGWSISAIAV<br>SQEHGLGLLDHPVSLATLKSFFITVNLAYSILKTEIAVVEVFLYNYLKMSVEATVELAVDTIDFTVVDERNQTVEDRTKKVSVGTDSVKKLTFLLKPNRSGNLPLT<br>VSAKSASERDSVQKILRVRSGGIQYYRNEARFIEVDGSTQNFNDIQLVIPRPATPGTENITFSVEGILLGAALTNLNKLIRLPTGCGEQTMLNLVPSVLALEYMTGT<br>GTLPDASKSQALDLLQKGYQNMLKYKLRDGSFSVFGQSDGRGSVFLTAFVAKTFRLAGKHITVDSGVVKTAYDWLAKQQETSGRFAELGKIWHQGIQGGLTGG<br>VPLTAYTLVAFLEQKNLVQQYKPVVDKGVAFLASKINELKKPYELALVSYALQLANHPQKGPALDKLLLEGHRSDTNSSTRWWDDGTTSIETTAYALLTFMNR<br>AMYVDAKPIMKWLVSQRYDKGGYGNTQNTFVGLQALADYSGKLSPSQNNYEIVISSSSGHRQTLSVNPQSSLISQRLTLPSSTRKVNVAITGTGTGVFQVAYQFN<br>APPTDAEPRFEIVKTQQETDTAVNLNICARYKPKKQDEVTNMVLMEVLFPSGYVVADETLNRLKSNKLVRKIETKRDETRLVLYFDSLPTEKATCVDVSGLRKAI<br>VLGQIAGLIKVYDYYEPVRGGMVMLSAL
DmTEP4/<br>FBgn004118<br>0	MEPVKAEGKYTIVGPGTIHSHRDYNVAVAVHQTKEPVTLKVGITGPSYNKTETVELATAGEFKQITFKLPPLEAGEYNLTAEGVKGLEFKNSTKLNWENFKPYIK<br>IQTDKGKYKPGDTINYRVIFLDENLRPDTAKDEVVVWFEDSKRNRIKQEKHIKTTGGVYTGKFELSEFATLGSWSLHVQNGDQHHDGGIYFGGRKQFGGFGHR<br>WHRSDELVNFEVEKYVLPKYSVKMDATQQVSVRDGEFNVVLKANYTYGKPVNGKVLVNVHLDSTSSWENVDGKTVQTDYPGHSVVGTADMVGGKAKLTM<br>DLKDFASYLPHKTSSSYAQITATVEEDFTGVKLNETGGVQLYPYRYEMSCTDYSSCFSFKPDKEHELNFKITYVDGSLITDTKSVVKAKFTEGIRRNYAFYAFGTD<br>HQEPELPTIEKKTFVFESHLNASGVAPFKVVLPDLPDIANFTRYYSIELEFVDEKRDLYTTYPYREPKQIENPSSEEEKEWFRAEVQRPKDVWNLKIGQEYQVILNS<br>SRPLKYFVYNIVGRGNILETKRVDLAEPQTTVNVTIKPTFLTTPYGRVYFYYVDETGEFRYTEETFSVEVELQNQIEIKAPAEVKPGADVALEIKTSPKSFVGLLAV<br>DQSVLLLGSNNDLNKESFNWRLNGYDTSTPWQGGYSYYPGERTGVVTMTNAYFFYNRTAPDYNILTEGFGGSSFAMRKTTVAHDSHVFHSGAGGPTQAVGFS<br>AESASASAAPVVRKNFAETWIFADIESTEEEVFKWVKTIPDTITNWVVTGFSLHPQKGLGVTNDQTNIKTFQPFFVSVRLPYSVKRGEVINVPALVFNYLPKTLDV<br>ELTLDNEDQEYDFVDASNEVIGDQKRTQNIRVGANEAAGASFLIRPKVIGNILLKFKAISPLAGDAIHKPLKVVPEGITQYQNRAFFINLKDTGEFKNTFELEVPED<br>VVPDSERVEFGLVGDLLGPVVKNLENLLRLPSGCGEQTMSKLVPNYLVRDYLKSIKKLTPALDTRIKRNLQDGYQHMLHYRHDDGSFSSFGPTKWRQEDPVRN<br>GSTWLTAYVLRSFSKIKDIIDLDEQILAKGYEFLLTRQAENGSFTEHGEYFYSSQRSLLTLTANSLLALLEEEKPNQAAIDKAVAYLSANTAESIELLPKSIAIYALQ<br>KAKAPEAAKQVASLKSLAKHEDDRTWWTEDLDKLRASKNCGRWWCWIWSQDVEITSYALLSLLDSDQETADSVLNTVRWLIAQRNGFGGFASSQDTVVGLT<br>ALIKFAEKSGYEAAKWEVTVSNKGKREKTEKLNTSEENDLLLQTVEFPQGTKSLEFEAKGTGAAMVQISYQYNLVEKEPKPSFKIQTTVLPESSPANLELSVCVD<br>YVEEGESKESNMAILEVSLPSGYTADEDSFADIRNIERVRLVETKNGDSVVVIYFENLAKNEEKCIRIEAYRTHAVANQKPSSVVLYDYYDTNKKATEYYSIKSKL<br>CDICEGDDCKSKC
DmTEP3/<br>FBgn004118<br>1	MRLQGADMGAIPVLILVTACLLCQTSAQGLYSIIAPNTLRPNSQFHVAVSLHNAPESATFKVGILGSSYTDFKTVELRPFSTQLLHFEIPALRTDRYRLTAEGLGGV<br>QFTNETQLHFESKQHTVLVQTDKSIYKPGDLVHYRVLILDANLKPARGYGRVHVDIKDSGDNIIRSYKDIRLTNSIYSNELRLSDSPRFGTWSIVVDVSDQEHTQTF<br>EILDHILPKFVVDIDTPKHAIYKDGKIAATVRAHYAFGQPIVGEATLSIYPTFFGSLQPFVNDLITRKVVPIDGNAYFEFDIENELHLKQDYERQYLLDALVEEKSTG<br>SVQNYSTVLTLHLNHYRVEAVKVPSYYIPGVPFEATARIARNDGGQLRDFNPQITAYLTNVYGSSEMYNRTAYSLDASGEIKMKFTVPIGDRDEFHSIIVDYQGVI<br>SEVGKIPSKHLHSKNYITAKVLNDRPTVNQEISVVVRSFAPIKYFMYQVVGRGDIILSRNVDVAPGTFHTIKFLASFAMMPRANLLVYTVIDGEFVYDEQVIQLEE<br>NLLNAVQVDAPIRAPPGQDIDIGISTKPYSYVGLMLVDQNANDLRSGHDLTHKRLMDALRSYELSDVNTPMGSPGKESGVITMSNTDYFIEKEAESNPALDREVS<br>TGPEEDKLTTVRKTDIGPAHKIEVNTLPPGKGRYAFSYTPKPFWHNPRVHVMRDPADTWLFLNISASSDGRNSIHRRIPSEMTSWVVSAFALDPVNGLGLSPPNG<br>KLEAYKEFYISTELPYSIKRDELIAIPFVVHNNRDSDLNVEVTFYNSALDFDFPQLDPKATNQPKVELYRRRSLQVPGRSARSVSFIVTPKRVGPLLVKAMAASSQA

	GDTVEQNLLVEHPGAMERINRGFLFELNSNAQNRRNVTIAVPRNAIPESTRIEVSAVGDLIGSLVGNLDSLILLPTGCGEQTMVNFVPNLIVLRYLGRLRQLTPEVE<br>LRATNNLAIGYQRILYYRHENGAFSAFGLDIKRSSTWLTAYVARSLRQAAPFTQVDSNVLQKALTYLGSVQSANGGFEERGDVFERFGDDGISLTAFVTLALMEN<br>VDLYPEYRNNINKALDFITRGLDGSSNLHAMAIGTYVLSRANHNAKAAFLQRLDSMATNKDGLKWWNKTAPAGEQQSPWYNATRSVNIEISAYAALALLENNL<br>VGDALPVLNWLMDQRNPKGGFVASQDTVVGLQALLMFAERFSSQGNNLQIGFHYGEGAETIINVNAENSLALQTVELPNNLKNLSVSATGRGMALAQVSYTYN<br>TNVTSAWPRFVLDPTVNRNSHADYLHLSACASFVSVVGENEQRSNMAVMEVHLPSGFVVDRDTLPTLESSERIKKVETQNRNTKVVIYFDYLDRREVCPTLHAY<br>KTVKVTKHRPVAVVMYDYYDSARRARQFYRAPKSNICDICEHANCGDLCEKAEKRESKRPDDYTAIAGHSSGSRHTAIPLASVVMVLSMLLKTLSC
DmTEP2/<br>FBgn004118<br>2	MFRIFLTGIILQYALLVNATGIYSVVGPGTLRSNSKYNVVVSVHKADGPSQIKVSLNGPSYNETKQIELPPMSTQNVEFEVPKLATGNYNLSAEGVSGVVFKNSTK<br>LNYADKKPSVFVQTDKATYKPADLVQFRILFLDENTRPAKIEKPISVIIIDGAQNRIKQLSDVKLTKGVFSGELQLSEQPVLGTWKISVSVDGDNRETKSFEVDKYV<br>LPKFEVIVDTPKAVVIADKVIKATIRAKYTYGKPVKGKATVSMERSYGYFGDLNANGNKQEKTIDVDGKGHVEFDIIHWAQRGQYLPPIKLFAVVTEELTGNKQ<br>NATATVVLHQQRYSIEPYERPEHFEANKSFIYQVVVKNVDGSPVTNSAKNVKIGFDKSYSYFHEPSPKTRINFEAPVNENGIATFNVRLPDSDSRYYRIFASFDGSE<br>NTIGSISKFEPTPMSREPLKIQVNTKKPRLGEQVSFDVVSIEDLPYFVYTIVARGNVILSDYVDVPDGQKTYTVKFTPTFSMVPKATIYVYYVVNNDLQFEEKTIDF<br>EKEFSNSIDVSAPTNAKPSEEVKLRIKTDADSFVGLLGVDQSVLLLKSGNDLSQDDIFNSLNIYQTSTPWMNGYGRYPGQTSGLVTLTNANYPYNTDSYSIYPLILN<br>GEFPIAFSLAAPQAAIAGMPGTSSIASHPNQAPQIRKEFPENWIFYNAENVGEEEFTLTKKIPDTITSWVVTGFSLNPTSGIALTKNPSKIRVFQPFFVSTNLPYSVKR<br>GEVIAIPVVIFNYLDKTLDADVVMDNSDQEYEFTEATNEVLEKAIDEVRRVKRVTIPANSGKSVSFMIRPKNVGFTTLKITATSALAGDAIHQKLKVEPEGVTLFE<br>NRAVFINLKDQPEMSQSLDADIPNEVVPQSEFIEFSVVGDLLGPTLQNLDNLVRMPYGCGEQNMVNFVPNILVLKYLEVTGRKLPSVESKARKFLEIGYQRELTY<br>KHDDGSYSAFGKSDASGSTWLTAYVMRSFHQAGTYTDIDPKVITAGLDFLVSKQKESGEFPEVGKLFDNANQNPLALTSFVLLAFFENHELIPKYQSAIKKAVRY<br>VAEEADKTDDQYSLAIAAVALQLAKHPQSEKVIAKLESVARKENDRMWWSKATESTGEDGRVFHWKPRSNDVEITSYVLLALLEKDPAEKALPIIKWLISQRNS<br>NGGFSSTQDTVIGLQALTKFAYKTGSGSGTMDIEFSSAGESKNTIKVNPENSLVLQTHDLPKSTRKVDFTAKGTGSAMVQLSYRYNLAEKEKKPSFKVTPTVKDT<br>PNQLLIVDVCAEYVPLEDADKDKDSNMAVMEIALPSGFVGDSTSLGKIQAVDRVKRVETKNSDSTVVVYFDSLTPGDVRCLPLEASKAHAVAKQKPASVSLYD<br>YYDTERKATEYYQVKSSLCDICEGADCGEGCKKD
DmTEP1/<br>FBgn004118<br>3	MLWLILSSTILHCVLLSNANGLYSVLAPKTLRSNSAYNVVVAIHNTTRTTEVSVSLTGPSLNSRKYVDVQSMSSKSVRFDIPKLTEGDYELKVMGSGGIEFQNSTK<br>LSFAPDLNWLYIQSDKATYKPGDKIQFRVLFLDKNTRPAVIDKPIKIEIRDGDQNLIKSWKDIKPAKGVYSGELQLSDRPVLGNWTVTATVQDEGKVTNVLVVDK<br>YVVPKFEVVVLTAKNVAASAGYIRATIKARYTFKKPVKGHVVATIEGSSTEQSLPIDGEVNVEFPISATAKRLLKITAIVTEELTDIKHNGTAYVTVHQHRHKLED<br>LFWPTHYRPGVSSEFKTVVRNLDGSPVMDSSKMVNFNVLCCQVSKNFSASLQNSIATEHIMLPETCQSCLVTSTFDTAENIERYIYKLNKPLMIAINTKKPQLRKL<br>LKINIISDTYLPYFILTVVARGNIVLSLFQEMKEKKKSQEIEFEPTFALVPQATIFVHYIIDGVLMSDEKTVDIERDFENTIEILTTNEALPRDEVSLKVKTNPHSFVGL<br>LGVDQSVLLLRSGNDLNRDLILNNLATYSTDLVILTNANINIYRSSGGCYTNPGYTNCTGSLIGRTMFKNEPTKNSGPVPIVGSTRAQASLPPVRKLFPETWLFSNIT<br>DVGANGEYIIKETVPDTLTSWVITGFSLSPQSGLAVTRNPSRIRVFQPFFITTNLPYSVKRGEVIAIPVIVFNYLGMDVKAKVLMDNSDGQYEFIETTNKNVSQYLR<br>GVRRKKTLWIPANTGRGISFMIRPKKVGLTTLKITAISKYAGDRLHQILKVEADGVQKYVNKAVLINVQRLNRRSLAPPEKTIIIEKADNVIEGSETVEFEVCGTSQ<br>APQLEHLDDLVHLPCGCGEQNMFNFVPSILALSYLKAKNRQDQEIENKAKRYVETGYQIELNYKRNDGSFSAWGQHDALGSTWLTAYVIRSFHQAAKYIDIDKN<br>VLVAGLDFLVSRQSTDGKFKELGMVIHNSHGSPLALTSFVLLTFFENEEYMPKYKHVIDRAVEFVVTEVHQSNEPYDLAIAALALSLARNRNAYKVLDKLDKLA<br>TRRGDHKWWTGSDKCKSSEVETTSYVLLALLEHNISDEPKPIVDWLISKRNSNGGFVSSQDTVVGIMALTKYELQSHASTEAIDIEFWHLNEDKKHVRVTKENEF<br>KVQTHQLPENTNEVKLLAKGQGRAQVQLTYRYNVATKEARPSFKLTTTVKKSHKGRLILGICGTYTPIAASERNKTTNMALMQVQLPSGYVCDIEPFADIEAISD<br>VKRVETKNEDTEVHIYFEKLSPGDRKCLTLEAIYTHAVANLKPSWVRLYDYYATERSATEFYHVDTSLCDICHGNECGNMC
DmTEP6/Dm<br>MCR/<br>FBgn026748<br>8	MMWHLLRALLVVAAVLDALQPAVGQNDNYYNPNQNQQNPQQPLLPNQQWGNNPQTNQYSNNNQNFGQTNPSDRPPYRTDSGSYNDIAGQDDYNKRVGGGY<br>QDNEEPSLTRGKSSYNIKATFLESLHSREPTYFIVASRMVRPGLIYQVSVSILQAQYPITVHASIACDGVQISGDSKDVKEGIPETLLMRIPPTSVTGSYKLRVEGFYQ<br>NVFGGLAFLNETRLDFSQRSMTIFVQTDKPLYMQGETVRFRTIPITTELKGFDNPVDVYMLDPNRHILKRWLSRQSNLGSVSLEYKLSDQPTFGEWTIRVIAQGQQ<br>EESHFTVEEYYQTRFEVNVTMPAYFFTTDPFIYGRVMANFTSGLPVRGNLTIKATIRPIGYFSNQVLNEKYRLGRSPLEQTNLYNERWRYNNPNQNPQVQYNVPG<br>QLPQDGADLSQDILYRNQYVVERHYQFDEEWPFWVRKPEYQDSSYEAWSGTYRKTLPYLRYFNGTFDFKWPLRELELLVPNLANSEVLITATVGEKFYDEIISG<br>YSVARVYNSSLRVVFLGDSPQVFKPAMPFTTYLAVEYHDGSPIDPNLLRQGLMEVSGFVESRNGGRRDWPAQRLPMSQQSDGIWEVKIDIRNDLNLDDRPQARD<br>FLNGVQNMRLQANFVDPRGERIQTELLLVSHYSPRNQHIKVTTSTEKPVVGEYIIFHIRTNFYLEEFNYLIMSKGVILVNDRETITEGIKTIAVVLSSEMAPVATIVV<br>WKINQQGQVVADSLTFPVNGISRNNFTVYINNRKARTGEKVEVAIFGEPGSYVGLSGIDSAFYTMQAGNELTYAKIITKMSNFDEQTNGTYKHIWYSHEGNPDEL<br>VYFPASSFGVDANRTFEYSGLIVFTDGYVPRRQDTCNRTLGFGECLSGRCYRLEKQCDGLFDCDDGTDEINCHARNDTELLNYRKYRFNRVLRHYENVWLWKD<br>VNIGPHGRYIFNVEVPDRPAYWMVSAFSVSPSKGFGMMNKALEYVGVQPFFINVEMPEACRQGEQVGIRVTVFNYMITPIEAIVVLHDSPDYKFVHVEEDGIVRS<br>YNPRTSFGEHQFFIYLEAQGTTVVYVPVVPQRLGNVDVTLHVATLLGTDTITRTLHVESDGLPQYRHQSVLLDLSNRAYVLEYMHVNVTQTPEIPYQVDRYFVY<br>GSNKARISVVGDVVGPIFPTMPVNASSLLSLPMESGEQNAFSFAANLYTIMYMRLINQRNKTLEKNAFYHMNIGYQRQLSFMRPDGSFSLFRSDWNNSDSSVWLT<br>SYCLRVFQEASFYEWENFIWIDATIIEKNMRWLLQHQTPQGSFFEVTWLPDRKMNRTNFDKNITLTSHVLITLATVKDISGTLGSRVALATQRALAYIERNMDFLR<br>HQAQPFDVAITAYALQLCNSPIAEEVFAILRRQARTIGDFMYWGNQEIPQPPRKLENQKWFSLPRLPYEYDSLNIETTAYALLVYVARREFFVDPIVRWLNSQRLN<br>DGGWASTQDTSAALKALVEYTVRSRLREVSSLTVEIEASSQGGKTQTLYIDDTNLAKLQSIEIPDAWGTIKVQAKGAGYAILQMHVQYNVDIEKFQTKPPVPAFG<br>LHTKAIFHGRNQSHISYVACQNWINQNESERSGMAVLDVAIPTGYWIQQQKLDTYVLSNRVRNLRRARYLERKIVFYFDYLDHEDICVNFTIERWYPVANMSRY<br>LPVRIYDYYAPERFNESIFDALPTYLLNICEVCGSSQCPYCSIYNMGWRASMSMSLLFFSVFIYLLRSRTHLVLNMMQLLT

Table S3. Effect sizes for main figure analyses.

Figure Number	GROUP	cliff DELTA	
figure1b	dsGFP vs dsTEP13	0.675	
figure1b	dsGFP vs dsTEP23b	0.648	
figure1b	dsGFP vs dsTEP27	0.818	
figure2a	dsGFP vs dsTEP27	0.4	
figure2b	dsGFP vs dsTEP27	0.748	
figure2c	1:10(preimmune:anti-CqTEP27)	0.694	
figure2c	1:100(preimmune:anti-CqTEP27)	0.692	
figure2c	1:1000(preimmune:anti-CqTEP27)	0.004	
figure2d	1:10(preimmune:anti-CqTEP27)	0.725	
figure2d	1:100(preimmune:anti-CqTEP27)	0.668	
figure2d	1:1000(preimmune:anti-CqTEP27)	0.122	
figure4b	untreated vs anti-tep27	0.444	
figure5c	CECA(preimmune:anti-CqTEP27)	0.778	
figure5c	CECA2(preimmune:anti-CqTEP27)	0.833	
figure5c	CECB(preimmune:anti-CqTEP27)	0.944	
figure5c	DEFA(preimmune:anti-CqTEP27)	0.611	
figure5c	DEFC-like(preimmune:anti-CqTEP27)	0.778	
figure5d	CECA(preimmune:anti-CqTEP27)	0.778	
figure5d	CECA2(preimmune:anti-CqTEP27)	0.833	
figure5d	CECB(preimmune:anti-CqTEP27)	0.944	
figure5d	DEFA(preimmune:anti-CqTEP27)	0.722	
figure5d	DEFC-like(preimmune:anti-CqTEP27)	0.611	
figure5e	dsGFP vs dstep27	0.799	
figure5e	dsGFP vs dsTEP27+dsceca	0.451	
figure5e	dsGFP vs dstep27+dsdefa	0.538	
figure5e	dsTEP27 vs dsCqTEP27+dsCECA	0.533	
figure5e	dsTEP27 vs dsCqTEP27+dsDEFA	0.359	
figure6d	BSA vs TEP27	0.083	
**Figure Number**	**GROUP**	**Odds ratio**	**95% CI**
figure3c	1:10(preimmune:anti-CqTEP27)	14.5	4.11 to 41.59
figure3c	1:100(preimmune:anti-CqTEP27)	10.29	3.179 to 32.22
figure3d	dsGFP vs dsTEP27	22.1	4.831 to 103.3
figure4c	untreated vs anti-tep27	4.5	1.666 to 11.02
figure6d	BSA vs CqTEP27	8.538	3.379 to 19.96
